# Herbivory-induced metabolic reprogramming in soybean cultivars contrasting in resistance to *Bemisia tabaci* MEAM1

**DOI:** 10.1093/jisesa/ieag081

**Published:** 2026-08-04

**Authors:** Adriele de Castro Ferreira, Maria Carolina Farias e Silva, Raylson Lopes da Silva, Matheus Monteiro de Santana, Daniel Marques Pacheco, Stelamaris de Oliveira Paula Marinho, Rafael de Souza Miranda, Jose Bruno Malaquias, Jenilton Gomes da Cunha, Bruno Ettore Pavan, Alexandre Igor Azevedo Pereira, Luciana Barboza Silva

**Affiliations:** Agricultural Sciences, Federal University of Piauí, Bom Jesus, Piauí, Brazil; Agricultural Sciences, Federal University of Piauí, Bom Jesus, Piauí, Brazil; Agricultural Sciences, Federal University of Piauí, Bom Jesus, Piauí, Brazil; Agricultural Sciences, Federal University of Piauí, Bom Jesus, Piauí, Brazil; Agricultural Sciences, Federal University of Piauí, Bom Jesus, Piauí, Brazil; Agricultural Sciences, Federal University of Piauí, Bom Jesus, Piauí, Brazil; Agricultural Sciences, Federal University of Piauí, Bom Jesus, Piauí, Brazil; Department of Crop Production and Environmental Sciences, Center for Agricultural Sciences, Federal University of Paraíba, Areia, Paraíba, Brazil; Agricultural Sciences, Federal University of Piauí, Bom Jesus, Piauí, Brazil; Department of Plant Science, Food Technology and Socioeconomics, School of Engineering, São Paulo State University (UNESP), Ilha Solteira, São Paulo, Brazil; Department of Agronomy, Instituto Federal de Educação, Ciência e Tecnologia Goiano—Campus Urutaí, Urutaí, Goiás, Brazil; Agricultural Sciences, Federal University of Piauí, Bom Jesus, Piauí, Brazil

**Keywords:** whitefly, resistance, metabolomics, herbivory

## Abstract

This study examined how herbivory-induced metabolic reprogramming in soybean alters foliar metabolic profiles in cultivars contrasting in resistance to the phloem-feeding insect *Bemisia tabaci* MEAM1, providing insights into plant responses associated with insect herbivory. Foliar metabolite profiles were compared between 2 contrasting genotypes, AS3810 (resistant) and BÔNUS (susceptible), under infested and non-infested conditions across 4 sampling scenarios integrating phenological stage and response time. Metabolic responses were characterized using gas chromatography–mass spectrometry (GC–MS) combined with univariate (3-way ANOVA) and multivariate analyses (principal component analysis, PCA). Significant main and interaction effects were detected for multiple metabolites, indicating that metabolic responses are strongly dependent on genotype and developmental context. Key compounds associated with treatment effects included salicylic acid, 4-aminobutyric acid (GABA), threonic acid, 2-oxoglutaric acid, glyoxylic acid, malic acid, spermidine, maltotriose, and galactonic acid, with several metabolites showing significant 3-way interactions. The resistant genotype AS3810 exhibited broader and more coordinated metabolic adjustments, particularly in pathways associated with redox balance, carbon allocation, and stress signaling, especially under reproductive-stage conditions. In contrast, BÔNUS showed lower metabolic responsiveness and reduced pathway integration. Multivariate analysis revealed metabolic differentiation among treatments, with defense- and stress-related metabolites contributing strongly to sample differentiation. These findings indicate that herbivory-induced metabolic configurations define distinct biochemical landscapes in host tissues and highlight genotype-dependent metabolic responses associated with herbivory. Identified metabolites represent candidate biochemical indicators of plant responses to *B. tabaci* MEAM1 infestation and may contribute to future studies of plant resistance mechanisms and resistance-based pest management strategies.

## Graphical AbstractGraphical Abstract

**Graphical Abstract:**
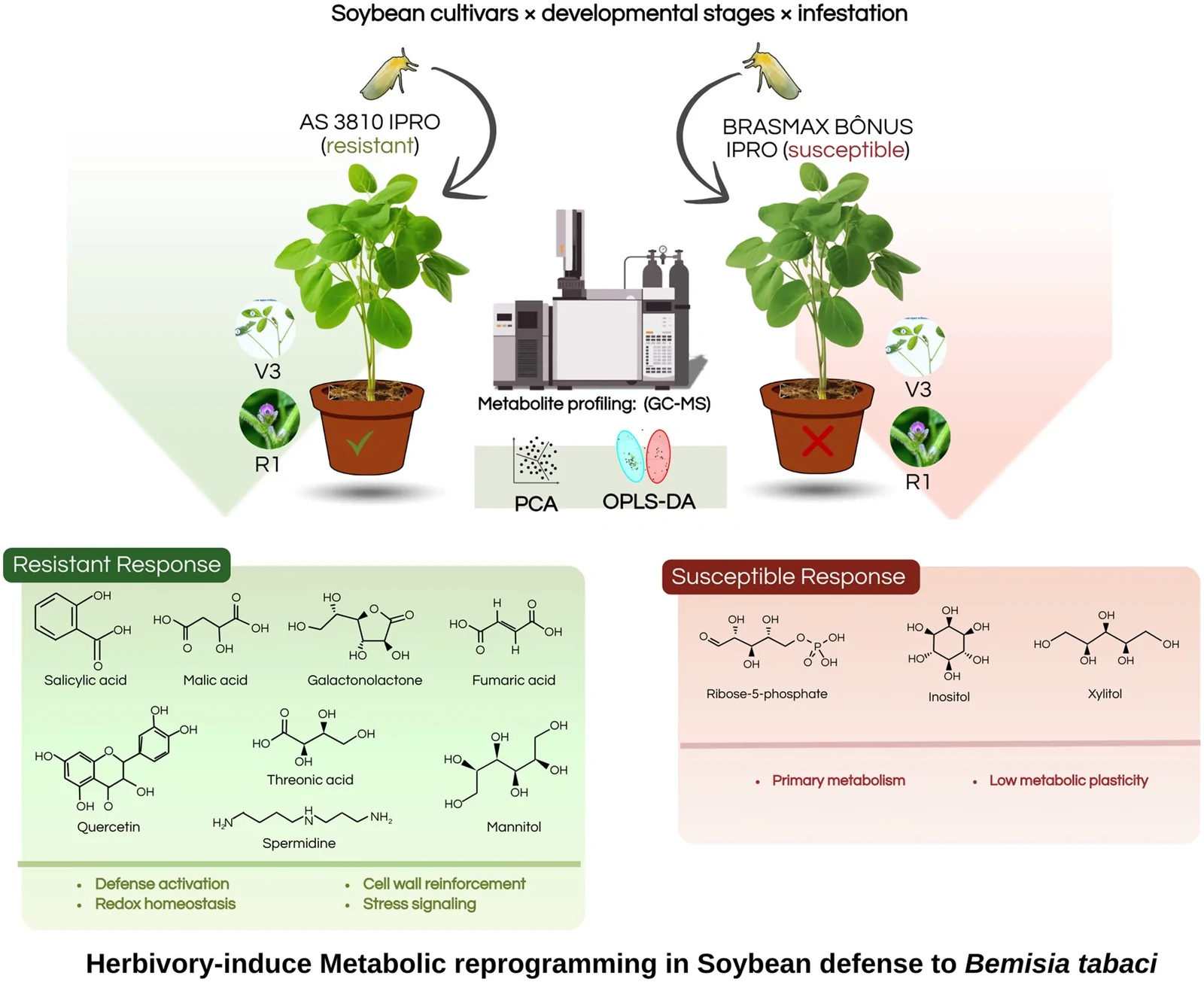

## Introduction

Soybean (*Glycine max* (L.) Merr.) is one of the most economically important crops worldwide due to its high protein and oil content and its extensive use in food, feed, and biofuel production (Yuan and Song 2023, Mishra et al. 2024). Brazil is currently the world’s leading soybean producer, and the crop plays a central role in national agricultural productivity, economic development, and food security. However, soybean production is significantly constrained by biotic stressors, particularly herbivorous insects such as the whitefly *Bemisia tabaci* (Gennadius 1889) MEAM1, a highly invasive and polyphagous species and an efficient vector of plant viruses (Fernandes et al. 2024, Nogueira et al. 2024, Silva et al. 2025).

Whitefly infestation causes direct physiological damage through phloem feeding, leading to reductions in photosynthetic efficiency, disruption of source–sink relationships, and metabolic imbalances. These plant responses affect host physiology and can substantially alter plant biochemical composition. For phloem-feeding insects, which rely on host-derived assimilates, herbivory-induced changes in plant metabolism may modify the nutritional and biochemical context of insect–plant interactions. Indirect effects through begomovirus transmission further exacerbate plant stress and contribute to complex physiological responses in host plants (Naveed et al. 2023, Idrees et al. 2024). In this context, host plant resistance is a key component of integrated pest management, providing a sustainable alternative to chemical control, which is often associated with resistance evolution and environmental risks (Gaddam et al. 2024).

From a physiological and ecological perspective, plant resistance to herbivory is expressed through dynamic biochemical adjustments that reshape plant responses to insect attack. Herbivory can trigger extensive metabolic reprogramming involving both primary and secondary metabolism, resulting in reallocation of resources toward defense, signaling, and cellular protection (Mostafa et al. 2022, Yash et al. 2025). These changes alter the chemical composition of plant tissues and may provide important indicators of defense-related responses.

Organic acids, phenolic compounds, amino acids, polyamines, and redox-related metabolites play central roles in these responses by regulating oxidative balance, structural defense, and signaling pathways (War et al. 2012, Raza et al. 2022, Gogoi et al. 2024). In phloem-feeding systems, changes in plant metabolite profiles have been associated with variation in nutritional quality, osmotic regulation, and defense-related processes, highlighting their importance in mediating plant responses to herbivory.

Recent advances in plant metabolomics have improved our understanding of genotype-specific responses to herbivory. Genotypes previously characterized as resistant often exhibit broader and more coordinated metabolic adjustments than susceptible genotypes (Erb and Kliebenstein 2020, Li et al. 2025a, 2025b). Importantly, these responses are frequently modulated by developmental stage, reflecting ontogenetic shifts in growth–defense trade-offs and contributing to variation in plant defense expression throughout development.

Despite these advances, integrative analyses investigating herbivory-induced metabolic reprogramming in soybean cultivars contrasting in resistance to *B. tabaci* across developmental stages and response times remain limited. This gap constrains our understanding of the biochemical mechanisms associated with plant responses to herbivory and limits the application of metabolomic information in resistance breeding and pest management strategies (Fraser and Chapple 2011, Obata and Fernie 2012).

We hypothesized that soybean cultivars previously characterized as contrasting in resistance to *B. tabaci* MEAM1 exhibit distinct metabolic configurations under infestation. Specifically, we expected the cultivar previously characterized as resistant to display broader and stage-dependent modulation of metabolites associated with redox balance, carbon metabolism, and defense signaling.

Therefore, the objective of this study was to characterize herbivory-induced metabolic reprogramming in soybean cultivars with contrasting resistance profiles and to identify metabolic responses associated with infestation by *B. tabaci* MEAM1. Using GC–MS-based metabolomic profiling, we compared foliar metabolic responses under infested and non-infested conditions at vegetative (V3, third trifoliate stage) and reproductive (R1, beginning flowering stage) developmental stages across defined response intervals. By integrating univariate and multivariate analyses, we aimed to identify metabolites associated with genotype-dependent responses to herbivory and to provide insights into biochemical processes potentially involved in soybean resistance mechanisms.

## Materials and Methods

### Experimental Conditions

The experiments were conducted in a greenhouse under controlled conditions (28 ± 3 °C; 50% to 60% relative humidity; photoperiod of 13:11 h L:D) at the Federal University of Piauí, Professora Cinobelina Elvas Campus (9°05′04.4″ S; 44°19′37.5″ W; altitude 270 m).

### Establishment and Maintenance of the *B. tabaci* MEAM1 Population

The *B. tabaci* MEAM1 population was originally obtained from a pesticide-free collard greens [*Brassica oleracea* (L.) var. sabellica] crop and maintained in a greenhouse covered with anti-aphid netting (50 mesh). Collard greens grown in five-liter pots were used as host plants for colony maintenance.

The identity of the whitefly population was molecularly confirmed as *B. tabaci* Middle East–Asia Minor 1 (MEAM1) by Dr. Eliane Dias Quintela (Embrapa Rice and Beans, Santo Antônio de Goiás, GO, Brazil). Adult specimens collected from the colony were subjected to molecular diagnosis based on mitochondrial cytochrome oxidase I (mtCOI) markers, following established protocols for cryptic species identification within the *B. tabaci* complex (Boykin et al. 2007, De Barro et al. 2011). The analyzed population was confirmed as belonging to the MEAM1 species, which is the predominant *B. tabaci* species associated with soybean production systems in Brazil.

To preserve genetic identity and colony stability, the population was maintained under greenhouse conditions without exposure to insecticides and periodically renewed with individuals from the original stock colony.

### Soybean Cultivars

The soybean cultivars used in the experiments were selected from those commonly cultivated by local producers and well adapted to regional environmental conditions. Two soybean cultivars previously characterized as contrasting in their responses to *B. tabaci* MEAM1 were selected for comparison. The cultivar AS3810 IPRO has been previously reported as exhibiting resistance-associated traits, including antibiosis against *B. tabaci* MEAM1, whereas the cultivar BRASMAX BÔNUS IPRO has been previously characterized as susceptible and highly attractive for adult landing, oviposition, and colonization by *B. tabaci* MEAM1 (Rodrigues et al. 2021, Silva et al. 2023).

### Experimental Procedure

Soil chemical properties were determined to guide liming and fertilization recommendations for soybean cultivation. Plants were grown in pots containing 10 kg of substrate composed of soil and cattle manure at a 3:1 ratio.

Five seeds were sown per pot, previously treated with insecticide and fungicide (Belure + Vitavax-Thiram 200 SC) and inoculated with Rhizobium spp. After emergence, thinning was performed to maintain 1 plant per pot. Irrigation was carried out according to plant water requirements, with watering directed to the base of the plant to avoid wetting the leaf surface and reduce the risk of phytopathogen development. Six plants per cultivar were used, with 3 plants infested with whiteflies and 3 maintained uninfested.

Three plants of each cultivar at the V3 (third trifoliate) and R1 (beginning flowering) phenological stages were infested with whiteflies. In each plant, 1 leaf was enclosed in cages made of voile fabric (13 cm × 13 cm), fixed to the petiole with satin ribbon, and containing 20 unsexed *B. tabaci* MEAM1 adults obtained from the laboratory colony.

Whiteflies were collected using an entomological aspirator (11 cm × 4 cm), preferentially selecting paired individuals, as whiteflies tend to remain coupled (Byrne et al. 1990). After 24 h of infestation (early response), the same procedure was applied to plants at the R1 stage to compare responses between vegetative and reproductive phases. Non-infested plants were used as controls. Successful infestation was confirmed by direct visual observation of adult survival and feeding activity within the cages throughout the infestation period.

A second experiment evaluating late responses was conducted to compare defense-related metabolic changes between cultivars previously characterized as resistant and susceptible to *B. tabaci* MEAM1. Plants infested at the V3 stage were evaluated after 30 d of infestation, whereas plants infested at the R1 stage were evaluated after 15 d. The same infestation methodology described for the early response experiment was applied.

At the end of each sampling period, the penultimate trifoliate leaf of each plant (from base to apex) was collected, immediately frozen in liquid nitrogen, and stored at −80 °C (Marchi-Werle et al. 2014) until analysis.

### GC–MS Metabolite Profiling and Data Analysis

Leaf samples were collected between 10:00 and 11:00 h. Plant material was immediately frozen in liquid nitrogen and stored at −80 °C until processing. Polar metabolite extraction was performed following Lisec et al. (2006), with minor modifications.

Fifty milligrams of powdered tissue were extracted with a methanol:chloroform:water solution (2:1:2, v/v/v). An aliquot of 150 µL from the upper (polar) methanol–water phase was transferred to a new tube and dried under vacuum at room temperature.

For derivatization, dried samples were treated with methoxyamine hydrochloride (20 mg mL^−1^) in anhydrous pyridine and incubated at 37 °C for 2 h, followed by the addition of N-methyl-N-(trimethylsilyl) trifluoroacetamide (MSTFA) and further incubation at 37 °C for 30 min.

Metabolite profiling was performed using gas chromatography–mass spectrometry (GC–MS; QP-PLUS 2010, Shimadzu, Japan). One microliter of each sample was injected in split mode (1:5). Helium was used as the carrier gas at a flow rate of 1.2 mL min^−1^. Metabolites were separated using an RTX-5MS capillary column (30 m × 0.25 mm × 0.25 µm) with an initial temperature of 80 °C for 2 min, increased at 10 °C min^−1^ to 315 °C, and held for 8 min.

Injector and ion source temperatures were maintained at 250 °C, and the MS interface temperature was set at 230 °C. The mass spectrometer operated in electron impact mode at 70 eV, with a scan range of 40 to 700 m/z, starting after a solvent delay of 3 min.

Chromatograms and mass spectra were processed using Xcalibur 2.1 software. Metabolite identification was performed by comparing mass spectra and retention characteristics with entries available in commercial mass spectral libraries and published reference datasets. Only metabolites showing consistent spectral matching and chromatographic quality were retained for subsequent analyses.

Files containing relative metabolite abundances from infested and non-infested soybean leaves were uploaded to the MetaboAnalyst 4.0 platform (http://www.metaboanalyst.ca) for subsequent analyses. For multivariate analyses, metabolite data were normalized, mean-centered, and Pareto-scaled prior to statistical evaluation.

### Experimental Design and Statistical Analyses

The experiment followed a completely randomized design with 2 soybean cultivars (AS3810 IPRO and BRASMAX BÔNUS IPRO) and 2 infestation conditions (infested with *B. tabaci* MEAM1 and non-infested control). Plants were evaluated at 2 phenological stages (V3, vegetative; R1, reproductive) under 2 response times: 24 h (early response) at both stages, and late response at 30 d for V3 and 15 d for R1. Because late-response evaluations differed between developmental stages, phenological stage and response time were combined into a single four-level factor (sampling condition) to represent biologically relevant infestation scenarios rather than independent temporal effects. Accordingly, sampling condition comprised 4 levels: V3–24 h, V3–30 d, R1–24 h, and R1–15 d.

For each cultivar × infestation × sampling condition combination, 3 independent biological replicates were used, with 1 plant representing 1 experimental unit, totaling 48 experimental units. Although metabolomic studies frequently employ a limited number of biological replicates due to the analytical complexity and cost of GC–MS analyses, the experimental design allowed the detection of biologically meaningful treatment effects and interactions.

For GC–MS metabolomic analysis, peak areas were normalized to the internal standard (ribitol) and sample fresh weight, and data were expressed as relative peak area. When necessary, data were cube-root transformed to meet model assumptions. Prior to multivariate analysis, data were Pareto-scaled.

For each metabolite, relative abundance was analyzed using 3-way analysis of variance (ANOVA) based on a linear model including fixed effects of Cultivar (2 levels), Infestation (2 levels), and Sampling condition (4 levels), as well as all 2- and 3-way interactions.

Assumptions of normality and homogeneity of variances were evaluated using the Shapiro–Wilk and Levene tests, respectively. When required, cube-root transformation was applied prior to analysis to meet model assumptions. Statistical significance was initially assessed at *P* ≤ 0.05, and false discovery rate (FDR) correction according to the Benjamini–Hochberg procedure was applied to account for multiple testing across metabolites.

When significant main effects or interactions were detected, interpretation focused on the magnitude and direction of effects across factor combinations, with particular attention to interaction terms. To facilitate biological interpretation of significant interactions, descriptive comparisons among treatment combinations were complemented by graphical visualization of selected metabolites showing strong treatment responses. Treatment effects were interpreted within the overall factorial framework and supported by multivariate analyses. All univariate analyses were performed in R (version 4.3.1; R Core Team 2023).

Multivariate analysis: Global metabolic variation among treatments was explored using Principal Component Analysis (PCA) based on mean-centered and Pareto-scaled data using the correlation matrix of variables. PCA was conducted in R using the prcomp function. Because the first 2 principal components explained a moderate proportion of total variance, PCA results were interpreted as indicators of metabolic differentiation patterns rather than complete separation among treatments.

A second PCA was performed using metabolites selected on the basis of their significant contributions to treatment discrimination, as identified from ANOVA results and PCA loading values. This approach was used to improve visualization of treatment-related metabolic patterns and identify metabolites contributing most strongly to sample differentiation.

The first 2 principal components were used to visualize metabolic differentiation among cultivars, infestation conditions, and sampling conditions.

## Results

### Herbivory-Induced Metabolic Reprogramming in Soybean Cultivars

GC–MS profiling detected 54 foliar metabolites encompassing organic acids, amino acids, sugars, polyols, phenolics, nucleotides, and polyamines. Three-way ANOVA demonstrated that metabolite abundance was significantly influenced by cultivar, infestation by *B. tabaci* MEAM1, sampling condition, and multiple interaction terms (Supplementary Table S1), indicating that metabolic responses depended on both genotype and developmental context.

Infestation exerted significant effects on a subset of metabolites, particularly organic acids, amino acids, and carbohydrates. Additionally, several metabolites exhibited significant cultivar × infestation and infestation × sampling condition interactions, indicating genotype-specific and stage-dependent metabolic modulation.

Overall, the resistant cultivar AS3810 IPRO exhibited a greater number of metabolites with significant responses and interaction effects across sampling conditions compared with BRASMAX BÔNUS IPRO. This pattern was especially evident under reproductive-stage sampling (R1–15 d), where multiple metabolites displayed significant interaction effects (Supplementary Tables S1 and S2).

### Differential Regulation of Defense-Related Metabolic Pathways

#### Organic Acids and Redox-Related Metabolites

Several organic acids were significantly influenced by infestation and interaction effects. Malonic acid, malic acid, glyoxylic acid, threonic acid, galactonic acid, and fumaric acid showed significant effects of sampling condition and/or infestation (Supplementary Table S1). Notably, galactonic acid exhibited significant cultivar and interaction effects, indicating genotype-dependent modulation.

Inspection of mean values (Supplementary Table S2) indicates that these metabolites exhibited condition-dependent variation across treatments. In AS3810, changes in malic acid, galactonic acid, threonic acid, and fumaric acid were observed under infestation, particularly during R1 sampling, although the direction and magnitude of responses varied across sampling conditions. Thus, under the R1–24 h sampling condition, infestation increased the levels of fumaric acid by 93.6%, malic acid by 10.8%, and galactonic acid by 1.5% compared to non-infested plants, whereas the level of threonic acid decreased by 47.6%. These metabolites are associated with central carbon metabolism, redox balance, and oxidative stress adjustment, consistent with metabolic reorganization in response to herbivory.

#### Amino Acids and Polyamines

Infestation significantly affected multiple amino acids, including threonine, serine, β-alanine, glycine, and aspartic acid, with significant main effects and interactions involving sampling condition (Supplementary Table S1). In the resistant cultivar AS3810 at the R1–24 h sampling condition, infestation increased threonine by 110.2% relative to non-infested plants. In contrast, serine, β-alanine, glycine, and aspartic acid decreased by 68.6%, 83.3%, 57.8%, and 79.7%, respectively (Supplementary Table S2).

Spermidine showed significant infestation and sampling effects, with descriptive patterns indicating treatment-dependent variation across sampling conditions. Increases in spermidine levels were observed in specific infestation scenarios, particularly under reproductive-stage conditions, although responses were not uniform across all treatments (Supplementary Table S2). This pattern is consistent with the involvement of nitrogen metabolism and polyamine-mediated stress signaling in herbivory responses.

4-Aminobutyric acid (GABA) also exhibited strong treatment-related variation, particularly across sampling conditions, reinforcing the role of stress-responsive amino acid pathways. Infestation resulted in a 43.6% reduction in GABA (4-aminobutyric acid) levels in the resistant cultivar AS3810 at the R1–24 h sampling condition compared with the corresponding non-infested plants (Supplementary Table S2).

#### Sugars and Polyols

Carbohydrates and polyols showed strong modulation by infestation and sampling condition. Maltotriose, isomaltose, maltose, ribose, ribose-5-phosphate, xylitol, rhamnose, mannitol, fructose, and glucose exhibited significant effects and/or interaction terms (Supplementary Table S1). In the resistant cultivar AS3810 at R1–15 d, infestation markedly altered carbon metabolism, increasing maltotriose, mannitol, glucose, ribose, and fructose levels by 76.7%, 72.8%, 64.0%, 61.5%, and 41.4%, respectively, compared with non-infested plants. In contrast, ribose-5-phosphate decreased by 35.7%, suggesting changes in carbon partitioning under herbivory (Supplementary Table S2).

In AS3810, several of these metabolites exhibited condition-dependent variation between infested and non-infested treatments, particularly at R1–15 d. In contrast, BRASMAX BÔNUS IPRO frequently showed overlapping values between infestation treatments, indicating comparatively lower metabolic responsiveness (Supplementary Table S2).

These patterns are consistent with reorganization of carbon allocation and osmoprotective metabolism under herbivory.

#### Phenolics and Antioxidant-Related Compounds

Phenolic metabolites were among the most responsive variables. Quercetin and salicylic acid showed significant effects of cultivar, infestation, and interaction terms (Supplementary Table S1).

Descriptive patterns in Supplementary Table S2 indicate treatment-dependent variation in these metabolites, with increases observed in specific infestation and sampling conditions, particularly during reproductive-stage sampling. However, responses were not uniform across all treatments.

Additional redox-related metabolites, including dehydroascorbic acid and erythronic acid derivatives, showed significant effects of sampling condition and/or interactions, indicating activation of oxidative stress-associated pathways. More specifically, under the R1–15 d sampling condition, infestation led to marked increases in the levels of erythronic acid-1,4-lactone, salicylic acid, quercetin, and dehydroascorbic acid, which were 93.0%, 64.0%, 44.3%, and 22.8% higher, respectively, than those observed in the corresponding non-infested AS3810 plants (Supplementary Table S2).

### Multivariate Discrimination and Identification of Metabolic Biomarkers

**Fig. 1.**
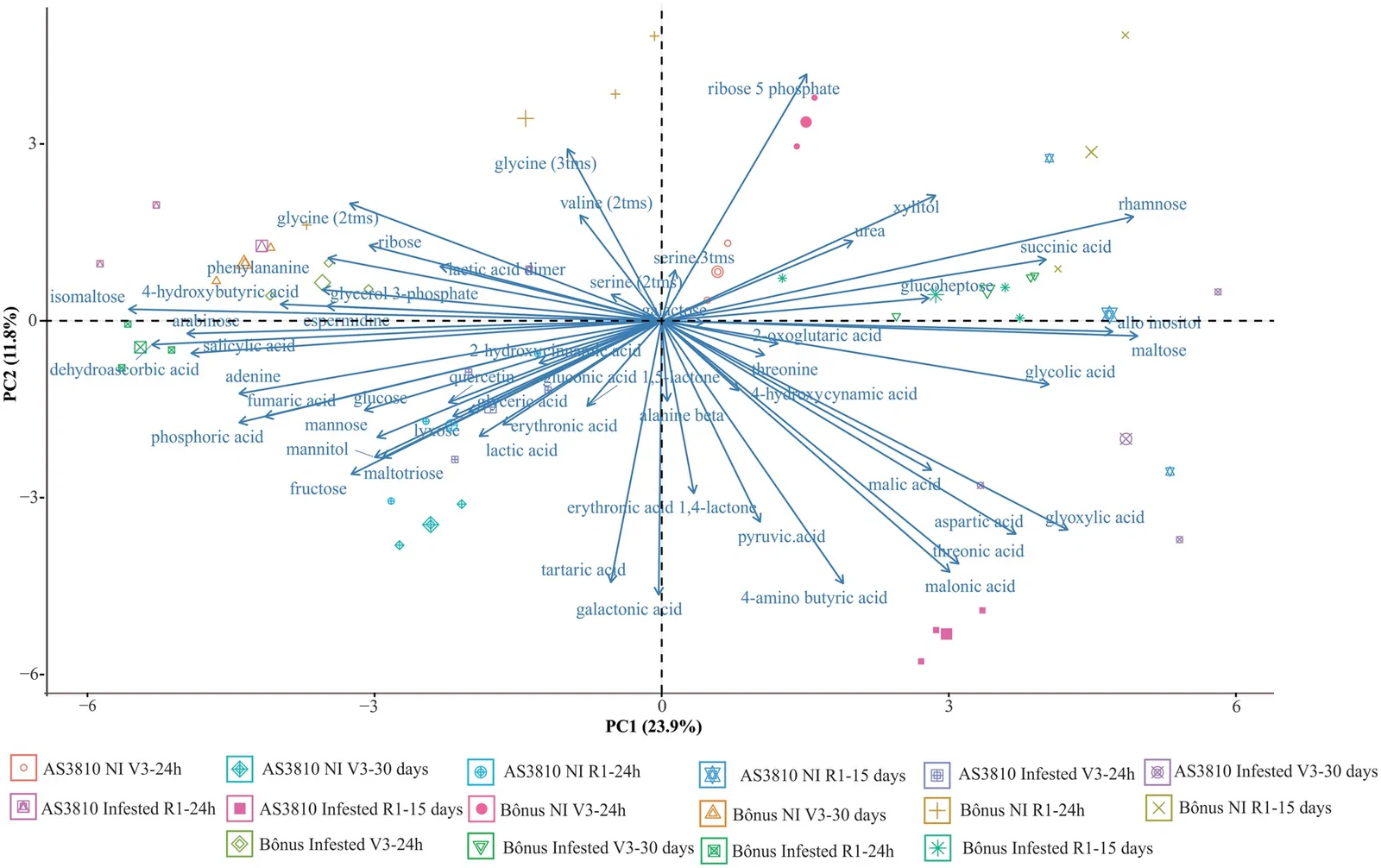
Principal component analysis (PCA) biplot of foliar metabolomic profiles of soybean cultivars AS3810 IPRO (resistant) and BRASMAX BÔNUS IPRO (susceptible) under infested and non-infested conditions by *Bemisia tabaci* MEAM1 across different sampling conditions (V3 and R1). The first 2 principal components (PC1 and PC2) explain 23.9% and 11.6% of the total variance, respectively. Points represent individual treatments, and vectors indicate metabolites contributing to group separation, with vector direction and length reflecting correlation and magnitude of contribution.

Principal component analysis (PCA) of all detected metabolites revealed structured metabolic segregation among cultivars, infestation treatments, and sampling conditions (Fig. 1).

In the global PCA, PC1 (23.9%) and PC2 (11.6%) together explained 35.5% of the total variance. PC1 separated samples associated with higher relative contributions of sugars and sugar alcohols (eg ribose-5-phosphate, xylitol, rhamnose, maltose) from those associated with organic acids and stress-related metabolites. PC2 captured variation related to sampling condition and treatment-dependent modulation.

Infested AS3810 samples, particularly at R1–15 d, were positioned in proximity to vectors corresponding to salicylic acid, quercetin, malic acid, fumaric acid, spermidine, galactonic acid, and threonic acid, indicating association with metabolites linked to redox balance and defense-related pathways (Fig. 1).

In contrast, BRASMAX BÔNUS IPRO samples, particularly under non-infested conditions, were more closely associated with vectors representing primary carbon metabolites such as ribose-5-phosphate, xylitol, rhamnose, and maltose.

**Fig. 2.**
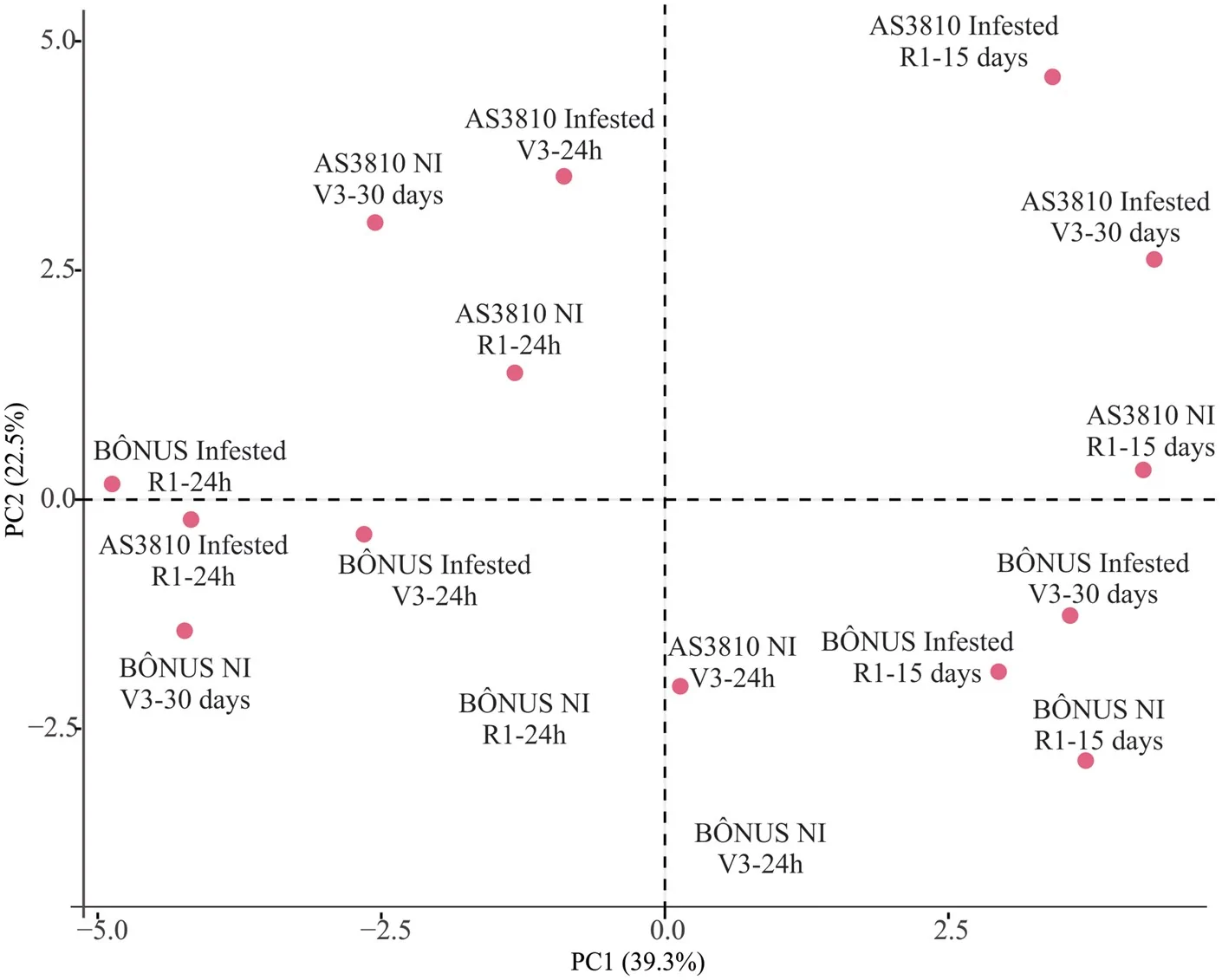
PCA scores plot based on selected foliar metabolites of soybean cultivars AS3810 IPRO (resistant) and BRASMAX BÔNUS IPRO (susceptible) subjected to infestation or non-infestation by *Bemisia tabaci* MEAM1 and evaluated at vegetative (V3) and reproductive (R1) stages. The first 2 principal components explain 39.3% (PC1) and 22.5% (PC2) of the total variance, respectively, totaling 61.8% of explained variance. Each point represents a specific treatment, and sample clustering reflects genotype-, infestation-, and sampling condition metabolic differences.

A refined PCA based on selected metabolites increased separation clarity, with PC1 (39.3%) and PC2 (22.5%) explaining 61.8% of the total variance (Fig. 2). This analysis indicated a clearer separation between infested and non-infested samples in AS3810, whereas BRASMAX BÔNUS IPRO showed partial overlap across treatments.

**Fig. 3.**
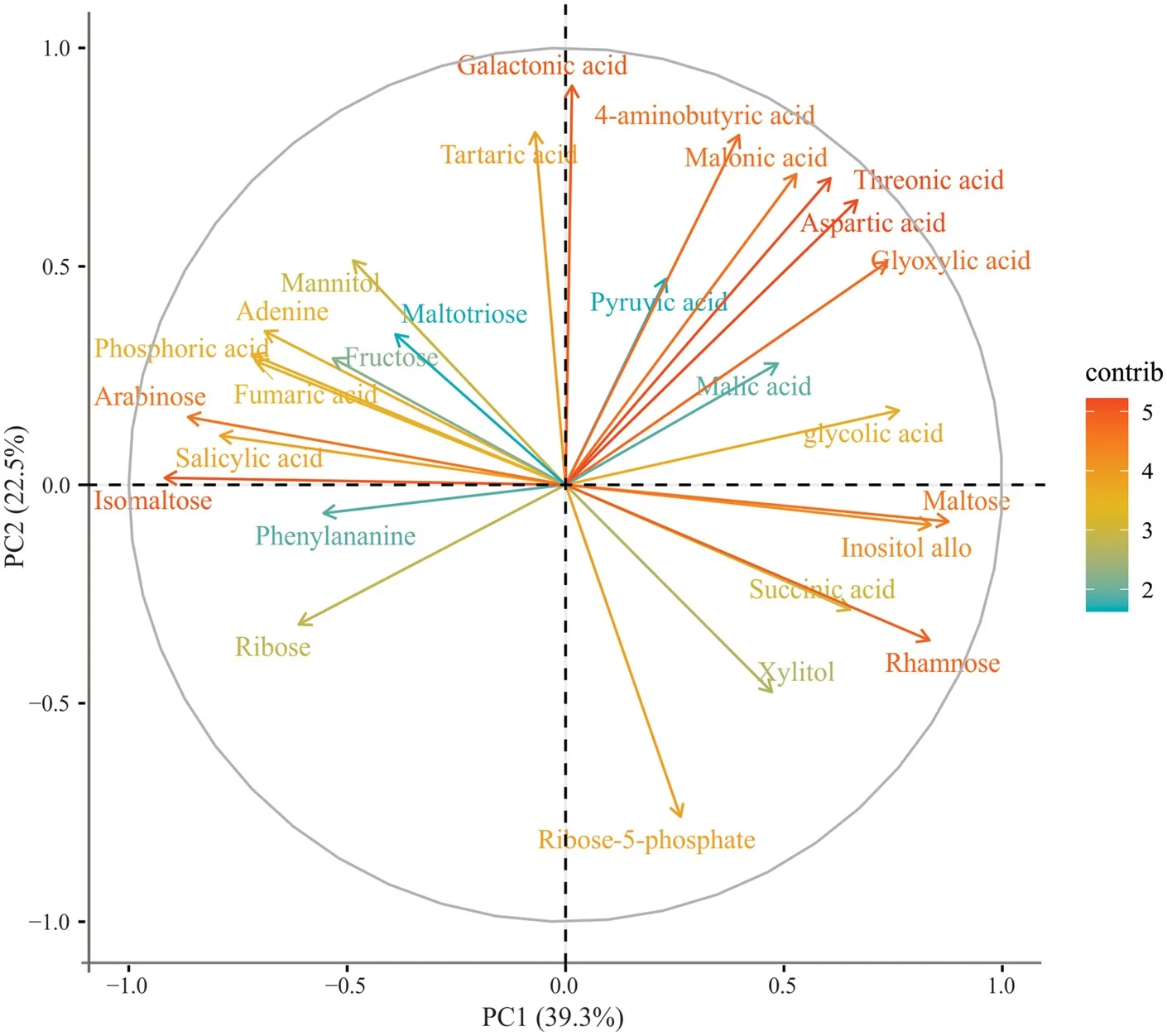
Contribution of individual metabolites to the first 2 principal components (PC1 and PC2) obtained from PCA of soybean foliar metabolomic profiles. PC1 and PC2 explain 39.3% and 22.5% of the total variance, respectively. Each vector represents a metabolite, with direction indicating correlation with the principal components and length reflecting its contribution to variation along principal components. Vector color denotes the relative contribution intensity (contrib), ranging from low (blue) to high (red).

Contribution analysis (Fig. 3) identified galactonic acid, threonic acid, glyoxylic acid, aspartic acid, salicylic acid, quercetin, and spermidine as major contributors to the variation observed along PC1 and PC2. These metabolites exhibited high loadings and were associated with sample distribution patterns related to infestation and cultivar.

Collectively, the results indicate that herbivory by *B. tabaci* MEAM1 is associated with metabolic reorganization in soybean leaves, which is genotype-dependent and influenced by developmental stage.

The resistant cultivar AS3810 exhibited a greater number of metabolites with significant responses and interaction effects, including organic acids, amino acids, polyamines, phenolics, and carbohydrates, particularly during reproductive-stage sampling. In contrast, BRASMAX BÔNUS IPRO showed comparatively lower differentiation between infested and non-infested conditions.

Metabolites such as salicylic acid, quercetin, spermidine, galactonic acid, malic acid, threonic acid, and selected carbohydrates were associated with variation in sample distribution and may represent candidate biochemical indicators linked to plant responses under herbivory by *B. tabaci* MEAM1.

## Discussion

The integration of univariate and multivariate analyses revealed clear genotype-dependent differences in metabolic responses to herbivory, resulting in distinct biochemical environments encountered by *B. tabaci* MEAM1 during feeding. These findings indicate that host metabolic configuration is not merely a plant response but a determinant of the nutritional and physiological landscape experienced by the insect. The significant main and interaction effects detected by the 3-way ANOVA demonstrate that these responses are highly context-dependent, varying according to both plant genotype and developmental stage. Similar patterns of genotype-dependent metabolic plasticity under herbivory have been reported in whitefly–tomato and aphid–Arabidopsis systems, where resistant genotypes exhibit stronger and more coordinated metabolic reprogramming (Casteel et al. 2014, Su et al. 2016).

The greater number of metabolites with significant responses observed in the resistant cultivar AS3810 IPRO suggests enhanced metabolic plasticity, which translates into a more heterogeneous and potentially restrictive biochemical environment for the insect. From an insect-centered perspective, such environments may impose constraints on feeding stability, nutrient acquisition, and metabolic homeostasis. This pattern was particularly evident under reproductive-stage conditions (R1–15 d), where interaction effects were most pronounced, indicating that ontogenetic stage modulates not only plant defense expression but also the degree of constraint imposed on herbivore performance.

Organic acids such as malic, galactonic, threonic, glyoxylic, and fumaric acids were strongly influenced by herbivory and interaction terms. These metabolites are closely associated with tricarboxylic acid (TCA) cycle dynamics, photorespiration, and redox buffering. Their modulation under infestation suggests a reorganization of carbon fluxes and energy metabolism that may alter the availability of metabolic intermediates essential for phloem-feeding insects. In systems involving *B. tabaci*, such changes have been linked to altered source–sink relationships and reduced feeding efficiency (Su et al. 2016, Perez-Fons et al. 2019). From a physiological standpoint, shifts in TCA-related metabolites may affect the balance between carbon supply and energy demand in the insect, potentially limiting assimilation efficiency.

The accumulation of galactonic and threonic acids, associated with ascorbate turnover, indicates enhanced antioxidant activity under herbivory. Whitefly feeding is known to induce reactive oxygen species (ROS) production in host tissues (Walling 2008), and the activation of antioxidant pathways reflects an active redox buffering response. For the insect, increased redox activity in host tissues may translate into a more chemically reactive feeding environment, potentially disrupting cellular homeostasis and affecting feeding persistence.

Salicylic acid (SA) showed strong modulation across treatments and is widely recognized as a central signaling molecule in plant defense against phloem-feeding insects. Elevated SA levels have been associated with reduced feeding efficiency and altered probing behavior in *B. tabaci* (Zarate et al. 2007, Walling 2008). The increased accumulation of SA in AS3810 IPRO under infestation suggests activation of phloem-based defense mechanisms that may directly interfere with insect feeding behavior through changes in sieve element chemistry and signaling dynamics.

Flavonoids such as quercetin also exhibited significant modulation, particularly in the resistant genotype. These compounds are known to act as antioxidants and anti-herbivore metabolites, affecting insect physiology through interference with digestion and detoxification pathways. The enhanced accumulation of quercetin is consistent with a biochemical environment that may reduce host suitability by increasing metabolic costs associated with detoxification.

Polyamine metabolism, particularly spermidine accumulation, showed strong treatment-dependent variation. Polyamines are multifunctional molecules involved in redox regulation, membrane stability, and signaling cross-talk. Their increased abundance under herbivory suggests a coordinated response linking nitrogen metabolism and oxidative balance. Experimental evidence indicates that elevated spermidine levels can negatively affect herbivore performance, potentially by altering cellular homeostasis or increasing oxidative stress (Qin et al. 2021). The coordinated modulation of spermidine and related pathways in AS3810 IPRO suggests a metabolic configuration that may impose additional physiological constraints on *B. tabaci*.

The strong variation observed in 4-aminobutyric acid (GABA) further supports this interpretation. The GABA shunt is a well-established stress-responsive pathway that is rapidly activated under biotic stress. Increased GABA levels have been shown to impair herbivore feeding and performance, likely through interference with neuromuscular signaling and metabolic processes (Bown et al. 2006, Scholz et al. 2015). The elevated GABA accumulation observed in the resistant genotype indicates activation of mechanisms that may directly affect insect physiology.

Carbohydrate metabolism was also substantially reorganized, with significant modulation of sugars and polyols such as maltotriose, isomaltose, ribose, ribose-5-phosphate, xylitol, and mannitol. Phloem-feeding insects depend on a precise osmotic balance for efficient feeding, and changes in sugar composition can directly affect ingestion rates and nutrient assimilation. Alterations in carbon allocation may therefore create osmotic constraints or imbalances that reduce feeding efficiency. Additionally, modulation of the pentose phosphate pathway, as indicated by ribose-5-phosphate variation, suggests increased demand for NADPH in redox defense, further reinforcing the link between metabolic reprogramming and oxidative stress.

The pronounced metabolic differentiation observed under reproductive-stage conditions highlights the importance of ontogeny in shaping plant–insect interactions. Increased defense investment during reproductive stages is consistent with optimal defense theory, where plants allocate resources to protect tissues with higher fitness value. In this context, the intensified metabolic response observed in AS3810 IPRO may represent a strategy that reduces host suitability during critical developmental phases, thereby limiting herbivore impact.

The PCA results reinforce these patterns by demonstrating coordinated metabolic shifts rather than isolated changes in individual pathways. The clustering of infested AS3810 samples with metabolites associated with defense and redox regulation supports the interpretation that resistance emerges from network-level reorganization of metabolism. This systems-level response is likely more effective in constraining herbivore performance than isolated biochemical changes.

Overall, the observed metabolic reprogramming defines distinct biochemical landscapes in host tissues that can impose multiple constraints on phloem-feeding insects, including nutrient imbalance, osmotic stress, and disruption of redox homeostasis. These constraints are likely to influence feeding behavior, physiological performance, and potentially adaptive responses of *B. tabaci*. The findings highlight that host suitability is an emergent property of integrated metabolic networks rather than individual compounds, providing important insights into the mechanistic basis of plant resistance and its ecological implications.

## Conclusion

Herbivory by *B. tabaci* MEAM1 induces genotype-dependent metabolic reprogramming in soybean, strongly modulated by developmental stage. The resistant cultivar AS3810 IPRO exhibited broader and more coordinated metabolic responses under infestation, particularly during reproductive-stage conditions, indicating enhanced metabolic plasticity.

These metabolic adjustments resulted in distinct biochemical environments in host tissues, reflecting coordinated changes in primary and secondary metabolism associated with the plant response to herbivory. Key metabolites, including salicylic acid, quercetin, spermidine, galactonic acid, malic acid, 4-aminobutyric acid, and threonic acid, were consistently associated with treatment-driven variation and are functionally linked to redox regulation, carbon allocation, and defense signaling pathways.

Multivariate analyses confirmed that these compounds contribute to the structuring of metabolic profiles across treatments, whereas the susceptible cultivar BRASMAX BÔNUS IPRO exhibited comparatively limited metabolic responsiveness and lower pathway coordination under herbivory.

Overall, the results demonstrate that soybean resistance to *B. tabaci* is associated with an integrated reconfiguration of metabolic networks involving carbon metabolism, amino acid metabolism, antioxidant responses, and defense-related pathways. These findings highlight that metabolic resistance emerges from integrated metabolic network responses rather than from isolated changes in individual metabolites and support the use of metabolite-based indicators in resistance breeding and ecologically informed pest management strategies.

## Supplementary Material

ieag081_Supplementary_Data
