# Supplementary material for: Herbivory-induced metabolic reprogramming in soybean cultivars contrasting in resistance to Bemisia tabaci MEAM1
Source: J Insect Sci. 2026 Aug 4;26(4):ieag081. doi: 10.1093/jisesa/ieag081 (PMC13563186; doi:10.1093/jisesa/ieag081)
Supplement: Supplementary file 1 [file ieag081_supplementary_data.zip › Supplementary Table S2 (2).docx]

Supplementary Table S2. Mean ± standard deviation (SD) of relative foliar metabolite abundance detected by GC–MS in soybean cultivars AS3810 IPRO (resistant) and BRASMAX BÔNUS IPRO (susceptible) under non-infested (Ni) and infested (I) conditions by *Bemisia tabaci* MEAM1 across four sampling conditions (V3–24 h, V3–30 d, R1–24 h, and R1–15 d). Values correspond to biological replicates (n = 3 per treatment combination) and were used for univariate (three-way ANOVA) and multivariate (PCA) analyses. Relative abundances were normalized to ribitol and fresh weight.

| **Metabolite** | **Cultivar - AS3810**  sampling conditions **V3 - 24** | | | | **Cultivar - BONUS**  sampling conditions **V3 - 24** | | | | **Cultivar - AS3810**  sampling conditions **R1 -24** | | | | **Cultivar - BONUS**  sampling conditions **R1 -24** | | | |
| --- | --- | --- | --- | --- | --- | --- | --- | --- | --- | --- | --- | --- | --- | --- | --- | --- |
|  | **Ni** | | | **I** | **Ni** | | **I** | | **Ni** | | **I** | | **Ni** | | | **I** |
| 1. Pyruvic acid | 2.30±0.10 | | | 1.89±0.31 | 1.30±0.53 | | 1.39±0.34 | | 1.45±1.43 | | 1.60±0.45 | | 2.53±0.22 | | | 1.57±0.36 |
| 2. Lactic acid | 4.60±0.49 | | | 6.99±0.82 | 4.38±0.48 | | 4.71±0.39 | | 3.28±1.36 | | 3.35±1.02 | | 4.04±0.77 | | | 5.50±1.83 |
| 3. Glycolic acid | 0.42±0.01 | | | 0.78±0.02 | 0.56±0.08 | | 0.59±0.06 | | 0.74±0.10 | | 0.60±0.09 | | 0.34±0.09 | | | 0.66±0.05 |
| 4. Malonic acid | 16.47±0.91 | | | 25.60±2.08 | 7.70±0.52 | | 12.43±0.78 | | 14.26±1.85 | | 16.08±1.23 | | 4.37±0.16 | | | 13.43±0.24 |
| 5. Valine (2TMS derivative)^1^ | 2.27±0.03 | | | 4.57±0.11 | 2.09±1.04 | | 4.70±0.92 | | 4.10±0.08 | | 23.90±7.02 | | 3.27±0.47 | | | 4.87±0.62 |
| 6. Urea | 1.24±0.21 | | | 4.83±0.83 | 2.18±1.27 | | 0.16±0.04 | | 0.28±0.04 | | 0.12±0.03 | | 3.40±0.13 | | | 0.23±0.01 |
| 7. Serine (2TMS derivative) | 19.64±0.31 | | | 23.93±1.86 | 27.19±5.10 | | 57.00±7.16 | | 49.55±0.93 | | 15.57±1.20 | | 29.09±1.23 | | | 16.05±4.33 |
| 8. Glycine | 4.55±0.87 | | | 5.84±0.08 | 3.31±0.45 | | 7.95±0.22 | | 7.90±0.53 | | 3.33±1.54 | | 7.35±0.06 | | | 10.08±0.28 |
| 9. Phosphoric acid | 69.25±26.66 | | | 115.53±16.86 | 66.86±40.55 | | 160.65±16.88 | | 194.09±0.25 | | 218.89±19.82 | | 203.74±34.94 | | | 328.80±8.12 |
| 10. Glyceric acid | 19.31±3.72 | | | 22.71±2.18 | 16.85±1.75 | | 29.62±1.24 | | 20.98±3.37 | | 18.80±0.49 | | 19.14±0.04 | | | 38.25±3.54 |
| 11. Fumaric acid | 41.00±5.53 | | | 34.57±0.49 | 23.73±0.38 | | 15.22±0.62 | | 25.93±0.80 | | 50.19±2.10 | | 7.17±1.12 | | | 43.44±1.21 |
| 12. Serine (3TMS derivative) | 8.65±1.90 | | | 20.21±2.75 | 8.45±4.39 | | 20.86±0.48 | | 20.34±2.34 | | 19.84±2.74 | | 19.69±0.86 | | | 10.35±0.68 |
| 13. Erythronic acid 1,4-lactone | 2.34±0.08 | | | 1.09±0.10 | 1.35±0.16 | | 1.31±0.06 | | 2.89±0.31 | | 1.16±0.08 | | 1.01±0.13 | | | 0.99±0.04 |
| 14. 4-Hydroxybutyric acid | 3.26±0.35 | | | 6.26±0.13 | 5.68±1.27 | | 4.64±0.31 | | 5.89±0.49 | | 4.17±0.35 | | 3.93±0.30 | | | 5.94±0.04 |
| 15. Threonine | 1.68±0.20 | | | 3.64±0.11 | 1.60±0.67 | | 2.80±0.08 | | 2.45±0.45 | | 5.15±0.54 | | 2.87±0.28 | | | 1.34±0.13 |
| 16. Glyoxylic acid | 1.57±0.07 | | | 1.93±0.16 | 1.65±0.14 | | 2.41±0.19 | | 0.93±0.18 | | 0.57±0.09 | | 0.90±0.16 | | | 0.84±0.08 |
| 17. β-Alanine | 1.99±0.48 | | | 0.52±0.05 | 0.99±0.45 | | 0.60±0.03 | | 2.21±0.09 | | 0.37±0.17 | | 1.27±0.05 | | | 0.31±0.03 |
| 18. Malic acid | 29.65±2.31 | | | 19.04±0.01 | 29.10±3.88 | | 18.19±0.83 | | 28.74±0.38 | | 31.83±2.44 | | 17.35±2.70 | | | 40.02±0.27 |
| 19. Salicylic acid | 1.05±0.17 | | | 0.76±0.02 | 0.44±0.05 | | 2.46±0.13 | | 1.94±0.14 | | 2.45±0.13 | | 0.96±0.12 | | | 5.80±0.19 |
| 20. Aspartic acid | 6.69±0.41 | | | 18.08±0.77 | 6.23±2.11 | | 7.11±0.03 | | 10.19±0.36 | | 2.07±0.23 | | 3.83±0.50 | | | 2.67±0.20 |
| 21. 4-Aminobutyric acid | 188.76±22.91 | | | 260.19±9.38 | 97.84±16.08 | | 243.06±21.47 | | 262.40±14.81 | | 148.01±16.21 | | 162.73±2.43 | | | 138.47±1.47 |
| 22. Erythronic acid | 0.60±0.07 | | | 0.52±0.02 | 0.42±0.04 | | 0.48±0.01 | | 0.89±0.07 | | 0.50±0.03 | | 0.45±0.02 | | | 0.88±0.05 |
| 23. Lactic acid dimer | 4.17±0.60 | | | 18.27±0.95 | 7.73±3.15 | | 9.23±0.17 | | 1.14±0.04 | | 8.44±0.98 | | 5.02±1.95 | | | 6.40±0.08 |
| 24. Threonic Acid | 2.76±0.04 | | | 7.42±0.64 | 3.31±0.52 | | 3.16±0.22 | | 5.67±0.14 | | 2.97±0.04 | | 3.62±0.12 | | | 4.25±0.04 |
| 25. 2-Oxoglutaric acid | 2.84±0.08 | | | 0.72±0.07 | 1.08±0.43 | | 0.71±0.02 | | 4.43±0.12 | | 0.62±0.19 | | 3.18±0.04 | | | 1.46±0.08 |
| 26. Phenylalanine | 5.34±0.32 | | | 9.28±0.39 | 7.59±2.86 | | 13.83±1.32 | | 11.22±1.96 | | 2.66±2.98 | | 9.22±3.84 | | | 3.34±0.98 |
| 27. Lyxose | 2.54±0.19 | | | 0.85±0.09 | 1.20±0.18 | | 1.30±0.16 | | 1.16±0.97 | | 1.80±0.28 | | 3.23±0.82 | | | 1.54±0.19 |
| 28. Arabinose | 2.22±0.08 | | | 2.76±0.08 | 2.61±0.27 | | 3.82±0.54 | | 2.61±0.26 | | 3.12±0.56 | | 2.81±0.22 | | | 3.72±0.18 |
| 29. Ribose | 5.88±0.40 | | | 0.84±0.09 | 4.21±0.05 | | 2.22±0.40 | | 2.61±0.72 | | 4.39±1.55 | | 7.94±2.20 | | | 5.20±0.69 |
| 30. Xylitol | 6.76±0.25 | | | 5.01±0.31 | 9.33±0.73 | | 7.20±0.57 | | 8.05±0.59 | | 6.14±0.17 | | 9.20±1.99 | | | 7.22±0.08 |
| 31. Rhamnose | 0.82±0.04 | | | 0.57±0.05 | 0.91±0.09 | | 0.67±0.04 | | 0.66±0.04 | | 0.73±0.03 | | 1.15±0.33 | | | 0.60±0.02 |
| 32. Glycerol-3-phosphate | 2.57±0.12 | | | 5.11±1.26 | 1.67±0.41 | | 4.95±0.92 | | 3.41±0.22 | | 1.56±4.24 | | 2.69±0.75 | | | 2.16±0.44 |
| 33. Tartaric acid | 3.48±0.07 | | | 4.90±0.61 | 3.23±0.67 | | 4.79±0.42 | | 5.04±1.54 | | 6.58±0.52 | | 3.53±0.91 | | | 5.91±0.56 |
| 34. 2-Hydroxycinnamic acid | 1.33±0.01 | | | 2.26±0.14 | 1.58±0.08 | | 3.25±0.41 | | 2.47±0.25 | | 3.89±0.54 | | 2.03±0.44 | | | 6.32±0.83 |
| 35. Adenine | 3.59±0.21 | | | 5.55±0.26 | 2.98±0.04 | | 6.45±0.47 | | 4.44±0.83 | | 2.20±1.20 | | 4.35±0.39 | | | 4.68±0.66 |
| 36. Fructose | 240.19±0.35 | | | 228.64±7.63 | 238.86±20.45 | | 265.15±13.99 | | 236.35±11.79 | | 243.21±4.34 | | 222.35±13.03 | | | 272.64±2.45 |
| 37. Galactose | 2.35±0.14 | | | 3.24±0.38 | 3.07±0.25 | | 3.41±0.23 | | 1.88±0.15 | | 4.07±0.72 | | 4.08±0.52 | | | 3.38±0.22 |
| 38. Mannose | 1.82±0.01 | | | 1.62±0.02 | 1.82±0.21 | | 2.05±0.08 | | 1.75±0.10 | | 1.89±0.07 | | 1.74±0.05 | | | 2.07±0.03 |
| 39. Glucose | 63.32±1.12 | | | 52.97±3.05 | 63.89±8.48 | | 72.27±3.41 | | 60.24±4.83 | | 67.67±4.08 | | 61.04±4.38 | | | 72.09±1.92 |
| 40. Mannitol | 5.08±0.36 | | | 7.81±1.57 | 1.31±0.20 | | 2.43±0.16 | | 9.82±0.63 | | 10.73±2.45 | | 1.99±0.31 | | | 3.08±0.20 |
| 41. Dehydroascorbic acid | 0.90±0.11 | | | 2.66±0.61 | 0.57±0.04 | | 2.83±0.48 | | 2.29±0.13 | | 1.33±1.12 | | 1.78±0.65 | | | 2.76±0.31 |
| 42. Allo-inositol | 36.40±1.94 | | | 33.98±4.35 | 52.43±4.24 | | 28.99±0.28 | | 27.52±1.46 | | 39.37±6.99 | | 40.52±8.63 | | | 31.73±2.85 |
| 43. Galactonic acid | 0.74±0.01 | | | 1.78±0.29 | 0.39±0.01 | | 0.88±0.15 | | 1.37±0.03 | | 1.39±0.13 | | 0.72±0.18 | | | 0.78±0.07 |
| 44. Gluconic Acid 1,5-lactone | 7.18±0.67 | | | 5.06±0.59 | 6.56±0.96 | | 9.18±0.85 | | 6.39±0.90 | | 3.03±0.11 | | 6.79±0.82 | | | 4.13±1.21 |
| 45. 4-Hydroxycinnamic acid | 0.59±0.05 | | | 0.75±0.09 | 0.54±0.08 | | 0.85±0.13 | | 0.89±0.02 | | 1.05±0.44 | | 0.65±0.11 | | | 1.25±0.11 |
| 46. Ribose-5-phosphate | 2.65±0.30 | | | 2.13±0.17 | 2.98±0.35 | | 2.95±0.35 | | 2.61±0.19 | | 2.12±0.76 | | 3.29±1.35 | | | 2.32±0.12 |
| 47. Glucoheptose | 0.49±0.03 | | | 0.45±0.04 | 0.57±0.04 | | 0.55±0.06 | | 0.54±0.03 | | 0.41±0.15 | | 0.51±0.04 | | | 0.39±0.02 |
| 48. Spermidine | 0.62±0.03 | | | 1.64±0.28 | 0.64±0.21 | | 2.91±0.29 | | 1.18±0.26 | | 0.37±0.26 | | 0.48±0.74 | | | 1.16±0.17 |
| 49. Isomaltose | 3.49±0.18 | | | 5.61±1.20 | 3.38±0.41 | | 5.54±0.43 | | 6.64±0.15 | | 6.13±3.26 | | 7.04±0.82 | | | 11.54±0.59 |
| 50. Maltose | 15.13±1.91 | | | 7.19±0.42 | 10.64±0.16 | | 9.25±0.73 | | 8.30±0.50 | | 8.81±1.95 | | 16.95±4.54 | | | 5.49±0.28 |
| 51. Quercetin | 62.73±4.95 | | | 81.34±19.18 | 45.22±3.99 | | 72.81±3.21 | | 76.07±3.88 | | 44.12±12.43 | | 57.94±5.89 | | | 63.19±6.95 |
| 52. Maltotriose | 47.42±7.62 | | | 127.97±17.41 | 65.94±2.99 | | 169.14±15.58 | | 65.51±9.93 | | 23.48±15.55 | | 58.36±28.46 | | | 127.25±28.66 |
| **Metabolite** | | **Cultivar - AS3810**  sampling conditions **V3 - 30** | | | | **Cultivar - BONUS**  sampling conditions **V3 - 30** | | | | **Cultivar - AS3810**  sampling conditions **R1 -15** | | | | **Cultivar - BONUS**  sampling conditions **R1 -15** | | |
|  |  | **Ni** | **I** | | | **Ni** | | **I** | | **Ni** | | **I** | | **Ni** | **I** | |
| 1. Pyruvic acid | | 3.87±0.18 | 2.30±0.99 | | | 3.12±0.45 | | 2.93±0.31 | | 4.60±0.08 | | 3.35±1.46 | | 1.37±0.21 | 0.88±0.57 | |
| 2. Lactic acid | | 11.78±0.95 | 4.32±0.49 | | | 5.65 ±1.13 | | 6.43±0.42 | | 6.14±0.07 | | 4.25±1.48 | | 6.46±0.03 | 6.31±1.13 | |
| 3. Glycolic acid | | 0.48±0.04 | 0.75±0.15 | | | 0.43±0.06 | | 0.78±0.08 | | 0.80±0.13 | | 0.81±0.08 | | 1.06±0.06 | 1.09±0.17 | |
| 4. Malonic acid | | 18.94±0.01 | 34.47±3.30 | | | 16.18±0.14 | | 13.16±0.05 | | 26.72±8.90 | | 39.09±3.91 | | 19.77±0.78 | 19.32±1.79 | |
| 5. Valine (2TMS derivative)^1^ | | 4.60±0.05 | 2.30±0.21 | | | 5.49±0.34 | | 2.73±0.02 | | 12.46±6.64 | | 2.34±0.29 | | 12.19±6.87 | 3.78±0.04 | |
| 6. Urea | | 5.69±0.05 | 2.18±0.38 | | | 0.10±0.01 | | 1.14±0.26 | | 1.91±0.09 | | 0.34±0.01 | | 9.02±4.58 | 2.58±0.10 | |
| 7. Serine (2TMS derivative) | | 26.49±0.85 | 16.81±0.60 | | | 21.57±1.32 | | 16.37±0.55 | | 22.20±2.55 | | 27.48±0.70 | | 40.31±6.70 | 38.24±0.16 | |
| 8. Glycine | | 6.15±0.11 | 2.54±0.07 | | | 11.10±0.11 | | 3.68±0.26 | | 3.91±1.21 | | 3.24±0.06 | | 10.04±4.70 | 4.01±0.46 | |
| 9. Phosphoric acid | | 164.41±4.26 | 128.22±15.31 | | | 279.37±1.45 | | 60.15±10.75 | | 59.80±20.28 | | 175.20±46.90 | | 81.90±0.19 | 104.53±16.22 | |
| 10. Glyceric acid | | 18.39±0.53 | 25.91±2.86 | | | 20.41±0.11 | | 24.91±2.56 | | 19.88±2.23 | | 20.34±0.22 | | 19.65±5.49 | 30.08±7.14 | |
| 11. Fumaric acid | | 43.56±0.49 | 22.19±1.31 | | | 48.52±1.97 | | 11.32±0.50 | | 14.95±4.24 | | 23.58±0.58 | | 1.57±0.38 | 6.37±1.16 | |
| 12. Serine (3TMS derivative) | | 17.12±0.66 | 19.06±1.71 | | | 15.36±1.92 | | 4.50±1.40 | | 14.57±2.86 | | 19.87±4.33 | | 37.34±13.80 | 16.61±0.56 | |
| 13. Erythronic acid 1,4-lactone | | 1.91±0.07 | 1.60±0.04 | | | 0.86±0.12 | | 1.36±0.18 | | 0.86±0.04 | | 1.66±0.21 | | 0.89±0.13 | 1.32±0.08 | |
| 14. 4-Hydroxybutyric acid | | 4.58±0.05 | 4.68±0.11 | | | 8.84±0.19 | | 3.17±0.45 | | 2.50±0.36 | | 1.88±0.03 | | 2.33±0.17 | 0.79±0.05 | |
| 15. Threonine | | 3.42±0.01 | 2.58±0.15 | | | 2.30±0.22 | | 3.06±0.67 | | 2.76±0.06 | | 5.17±0.12 | | 6.20±1.44 | 4.07±0.61 | |
| 16. Glyoxylic acid | | 2.27±0.17 | 4.36±0.57 | | | 0.35±0.03 | | 3.00±0.17 | | 2.48±0.48 | | 6.56±0.03 | | 2.86±0.23 | 3.40±0.22 | |
| 17. β-Alanine | | 1.31±0.01 | 0.35±0.09 | | | 0.39±0.01 | | 0.63±0.10 | | 0.75±0.02 | | 1.41±0.09 | | 0.41±0.08 | 1.16±0.02 | |
| 18. Malic acid | | 34.85±0.09 | 40.38±0.68 | | | 31.43±0.33 | | 28.70±1.52 | | 44.37±10.92 | | 49.40±3.29 | | 38.86±0.38 | 41.99±9.75 | |
| 19. Salicylic acid | | 1.78±0.01 | 0.44±0.02 | | | 2.35±0.03 | | 0.19±0.01 | | 0.50±0.09 | | 0.82±0.11 | | 0.28±0.06 | 0.48±0.04 | |
| 20. Aspartic acid | | 13.39±0.02 | 17.85±0.48 | | | 2.19±0.09 | | 12.77±1.24 | | 12.19±3.13 | | 18.78±1.03 | | 14.42±1.60 | 7.79±0.96 | |
| 21. 4-Aminobutyric acid | | 238.83±1.66 | 267.03±4.35 | | | 140.12±2.11 | | 225.45±13.61 | | 235.27±18.99 | | 274.75±18.50 | | 165.29±1.18 | 191.83±18.58 | |
| 22. Erythronic acid | | 0.44±0.29 | 0.62±0.04 | | | 0.69±0.01 | | 0.49±0.03 | | 0.65±0.07 | | 0.57±0.03 | | 0.40±0.03 | 0.41±0.06 | |
| 23. Lactic acid dimer | | 2.72±0.02 | 1.34±0.06 | | | 2.21±0.03 | | 1.91±0.10 | | 3.33±0.05 | | 1.35±0.03 | | 2.77±0.55 | 2.28±0.24 | |
| 24. Threonic Acid | | 4.76±0.08 | 7.81±1.81 | | | 2.19±0.10 | | 5.39±0.32 | | 5.26±1.40 | | 7.32±0.77 | | 5.51±0.32 | 4.77±1.11 | |
| 25. 2-Oxoglutaric acid | | 2.51±0.06 | 1.24±0.21 | | | 3.29±0.31 | | 1.60±0.21 | | 7.81±0.19 | | 1.84±0.02 | | 1.19±0.16 | 1.80±0.35 | |
| 26. Phenylalanine | | 7.40±0.09 | 3.11±0.63 | | | 6.39±0.10 | | 3.10±0.17 | | 5.53±1.66 | | 2.99±0.23 | | 4.75±0.85 | 4.92±0.63 | |
| 27. Lyxose | | 2.59±0.27 | 1.33±0.04 | | | 1.92±0.25 | | 1.18±0.18 | | 1.09±0.01 | | 1.55±0.26 | | 0.73±0.12 | 1.03±0.16 | |
| 28. Arabinose | | 3.66±0.08 | 2.17±0.09 | | | 3.40±0.02 | | 2.43±0.09 | | 1.94±0.13 | | 2.63±0.11 | | 2.52±0.15 | 2.63±0.10 | |
| 29. Ribose | | 4.08±0.20 | 1.91±1.25 | | | 8.75±0.78 | | 2.90±0.05 | | 1.74±0.11 | | 2.81±0.19 | | 1.58±0.29 | 3.32±0.83 | |
| 30. Xylitol | | 7.93±0.18 | 6.88±0.49 | | | 6.89±0.27 | | 9.55±0.60 | | 7.91±0.66 | | 7.49±0.17 | | 9.27±0.20 | 9.36±0.43 | |
| 31. Rhamnose | | 0.69±0.02 | 0.89±0.09 | | | 0.59±0.01 | | 1.40±0.20 | | 0.91±0.01 | | 1.08±0.10 | | 1.28±0.06 | 1.31±0.14 | |
| 32. Glycerol-3-phosphate | | 2.36±0.01 | 1.06±0.11 | | | 2.22±0.09 | | 1.40±0.20 | | 1.40±0.23 | | 2.20±0.08 | | 1.17±0.04 | 2.28±0.43 | |
| 33. Tartaric acid | | 6.59±0.01 | 6.07±1.04 | | | 4.47±0.34 | | 4.40±0.17 | | 5.57±0.92 | | 6.51±0.67 | | 4.30±0.17 | 4.06±0.87 | |
| 34. **Metabolite** | | **Cultivar - AS3810**  sampling conditions **V3 - 30** | | | | **Cultivar - BONUS**  sampling conditions **V3 - 30** | | | | **Cultivar - AS3810**  sampling conditions **R1 -15** | | | | **Cultivar - BONUS**  sampling conditions **R1 -15** | | |
| 35. | | **Ni** | **I** | | | **Ni** | | **I** | | **Ni** | | **I** | | **Ni** | **I** | |
| 36. 2-Hydroxycinnamic acid | | 2.72±0.01 | 3.49±0.83 | | | 5.48±0.01 | | 3.83±0.12 | | 3.33±0.69 | | 3.36±0.39 | | 4.28±0.19 | 3.74±0.52 | |
| 37. Adenine | | 4.39±0.10 | 2.95±0.62 | | | 4.73±0.02 | | 3.02±0.41 | | 3.37±0.15 | | 4.02±0.40 | | 3.63±0.46 | 2.75±0.33 | |
| 38. Fructose | | 281.69±1.94 | 235.73±12.85 | | | 262.33±3.01 | | 255.08±10.33 | | 196.80±9.48 | | 278.30±11.34 | | 206.78±21.43 | 255.47±11.05 | |
| 39. Galactose | | 3.42±0.10 | 2.31±0.20 | | | 0.02±0.01 | | 3.33±0.42 | | 2.96±0.05 | | 3.70±1.65 | | 1.47±0.98 | 5.08±2.17 | |
| 40. Mannose | | 2.23±0.02 | 1.82±0.15 | | | 2.03±0.04 | | 1.86±0.10 | | 1.42±0.05 | | 2.09±0.09 | | 1.57±0.19 | 2.01±0.08 | |
| 41. Glucose | | 75.97±0.64 | 61.86±8.61 | | | 68.81±1.36 | | 61.40±2.95 | | 44.13±2.14 | | 72.38±4.84 | | 50.39±9.19 | 71.56±2.47 | |
| 42. Mannitol | | 13.19±0.14 | 3.90±0.38 | | | 2.78±0.08 | | 1.94±0.52 | | 2.06±0.07 | | 3.56±0.99 | | 1.18±0.05 | 1.74±0.30 | |
| 43. Dehydroascorbic acid | | 2.63±0.05 | 0.80±0.29 | | | 1.79±0.06 | | 0.71±0.21 | | 0.79±0.02 | | 0.97±0.10 | | 0.93±0.11 | 0.71±0.12 | |
| 44. Allo-inositol | | 35.73±0.02 | 40.87±2.99 | | | 26.11±0.27 | | 49.51±2.02 | | 45.06±6.00 | | 50.96±1.75 | | 42.89±3.52 | 50.77±3.08 | |
| 45. Galactonic acid | | 1.76±0.03 | 1.73±0.22 | | | 0.69±0.01 | | 0.53±0.04 | | 1.38±0.57 | | 2.24±0.21 | | 0.70±0.08 | 0.73±0.14 | |
| 46. Gluconic Acid 1,5-lactone | | 9.86±0.21 | 6.21±1.70 | | | 4.28±0.14 | | 5.65±0.79 | | 3.95±0.05 | | 7.74±0.73 | | 4.96±1.23 | 9.33±0.32 | |
| 47. 4-Hydroxycinnamic acid | | 0.98±0.05 | 1.15±0.23 | | | 1.22±0.01 | | 1.19±0.05 | | 1.45±0.06 | | 1.38±0.19 | | 1.37±0.14 | 0.89±0.27 | |
| 48. Ribose-5-phosphate | | 1.49±0.01 | 1.88±0.62 | | | 2.49±0.01 | | 3.68±0.28 | | 3.47±0.55 | | 2.23±0.28 | | 3.43±0.05 | 3.94±0.42 | |
| 49. Glucoheptose | | 0.60±0.01 | 0.61±0.05 | | | 0.43±0.01 | | 0.69±0.05 | | 0.60±0.09 | | 0.59±0.03 | | 0.54±0.06 | 0.80±0.07 | |
| 50. Spermidine | | 0.19±0.02 | 0.18±0.04 | | | 0.15±0.02 | | 0.25±0.03 | | 0.26±0.04 | | 0.22±0.03 | | 0.33±0.01 | 0.36±0.05 | |
| 51. Isomaltose | | 8.29±0.06 | 2.15±0.37 | | | 10.97±0.01 | | 5.02±0.61 | | 2.44±0.63 | | 2.75±0.36 | | 1.82±0.54 | 3.75±0.68 | |
| 52. Maltose | | 6.64±0.30 | 12.28±5.31 | | | 4.16±0.07 | | 23.43±4.11 | | 14.96±0.59 | | 18.76±2.84 | | 12.16±4.26 | 20.88±1.51 | |
| 53. Quercetin | | 79.88±0.42 | 48.95±15.79 | | | 65.25±1.08 | | 85.69±14.00 | | 72.77±3.31 | | 104.98±26.82 | | 50.99±19.83 | 95.26±14.30 | |
| 54. Maltotriose | | 139.25±6.86 | 63.99±21.79 | | | 76.96±1.64 | | 63.88±23.05 | | 57.08±2.30 | | 100.87±9.46 | | 59.11±16.61 | 137.53±16.84 | |

^1^TMS = trimethylsilyl derivate.
