# Supplementary material for: Herbivory-induced metabolic reprogramming in soybean cultivars contrasting in resistance to Bemisia tabaci MEAM1
Source: J Insect Sci. 2026 Aug 4;26(4):ieag081. doi: 10.1093/jisesa/ieag081 (PMC13563186; doi:10.1093/jisesa/ieag081)
Supplement: Supplementary file 1 [file ieag081_supplementary_data.zip › Supplementary_Table_S1_JIS_54_metabolites - modificado.docx]

***Supplementary Table S1.*** *Three-way analysis of variance (ANOVA) of foliar metabolites detected by GC–MS in soybean cultivars AS3810 IPRO (resistant) and BRASMAX BÔNUS IPRO (susceptible) under infestation by Bemisia tabaci MEAM1. The statistical model included cultivar (2 levels), infestation (2 levels), and sampling condition (4 levels combining phenological stage and response time: V3–24 h, V3–30 d, R1–24 h, and R1–15 d), as well as all two- and three-way interactions. Mean squares, F values, p-values, and Benjamini–Hochberg false discovery rate (FDR) values are presented. Significance levels: p or FDR < 0.05 *, < 0.01 **, and < 0.001 ***; NS = not significant.*

| **Metabolite** | **Effect** | **df** | **Mean Square** | **F value** | **p-value** | **p significance** | **FDR (BH)** | **FDR significance** |
| --- | --- | --- | --- | --- | --- | --- | --- | --- |
| **Organic acids** | | | | | | | | |
| 2-Oxoglutaric acid | Cultivar | 1 | 11.1458 | 129.2900 | 0.0001 | *** | 0.0003 | *** |
|  | Infestation | 1 | 48.9042 | 567.2700 | 0.0001 | *** | 0.0003 | *** |
|  | Sampling condition | 3 | 6.8441 | 79.3900 | 0.0001 | *** | 0.0003 | *** |
|  | Cultivar × Infestation | 1 | 18.2164 | 211.3000 | 0.0001 | *** | 0.0003 | *** |
|  | Cultivar × Sampling condition | 3 | 8.1254 | 94.2500 | 0.0001 | *** | 0.0003 | *** |
|  | Sampling condition × Infestation | 3 | 2.0456 | 23.7300 | 0.0001 | *** | 0.0003 | *** |
|  | Cultivar × Sampling condition × Infestation | 3 | 6.9823 | 80.9900 | 0.0001 | *** | 0.0003 | *** |
| 4-Hydroxybutyric acid | Cultivar | 1 | 0.6769 | 1.7700 | 0.1927 | NS | 0.2658 | NS |
|  | Infestation | 1 | 2.4843 | 6.5000 | 0.0158 | * | 0.0337 | * |
|  | Sampling condition | 3 | 29.7863 | 77.9300 | 0.0001 | *** | 0.0003 | *** |
|  | Cultivar × Infestation | 1 | 11.3296 | 29.6400 | 0.0001 | *** | 0.0003 | *** |
|  | Cultivar × Sampling condition | 3 | 2.2214 | 5.8100 | 0.0027 | ** | 0.0070 | ** |
|  | Sampling condition × Infestation | 3 | 9.9081 | 25.9200 | 0.0001 | *** | 0.0003 | *** |
|  | Cultivar × Sampling condition × Infestation | 3 | 11.1318 | 29.1200 | 0.0001 | *** | 0.0003 | *** |
| Dehydroascorbic acid | Cultivar | 1 | 0.0036 | 0.0100 | 0.9129 | NS | 0.9301 | NS |
|  | Infestation | 1 | 0.5707 | 1.9000 | 0.1778 | NS | 0.2517 | NS |
|  | Sampling condition | 3 | 7.0245 | 23.4000 | 0.0001 | *** | 0.0003 | *** |
|  | Cultivar × Infestation | 1 | 0.1986 | 0.6600 | 0.4223 | NS | 0.4897 | NS |
|  | Cultivar × Sampling condition | 3 | 0.1705 | 0.5700 | 0.6402 | NS | 0.6954 | NS |
|  | Sampling condition × Infestation | 3 | 5.5272 | 18.4100 | 0.0001 | *** | 0.0003 | *** |
|  | Cultivar × Sampling condition × Infestation | 3 | 0.3762 | 1.2500 | 0.3075 | NS | 0.3871 | NS |
| Erythronic acid | Cultivar | 1 | 0.0397 | 2.5000 | 0.1237 | NS | 0.1893 | NS |
|  | Infestation | 1 | 0.0075 | 0.4700 | 0.4967 | NS | 0.5538 | NS |
|  | Sampling condition | 3 | 0.0731 | 4.6100 | 0.0087 | ** | 0.0193 | * |
|  | Cultivar × Infestation | 1 | 0.0850 | 5.3600 | 0.0272 | * | 0.0544 | NS |
|  | Cultivar × Sampling condition | 3 | 0.0525 | 3.3100 | 0.0323 | * | 0.0620 | NS |
|  | Sampling condition × Infestation | 3 | 0.0001 | 0.0000 | 0.9996 | NS | 0.9996 | NS |
|  | Cultivar × Sampling condition × Infestation | 3 | 0.1225 | 7.7200 | 0.0005 | *** | 0.0015 | ** |
| Erythronic acid 1,4-lactone | Cultivar | 1 | 4.0717 | 108.8900 | 0.0001 | *** | 0.0003 | *** |
|  | Infestation | 1 | 0.2437 | 6.5200 | 0.0157 | * | 0.0337 | * |
|  | Sampling condition | 3 | 0.1952 | 5.2200 | 0.0048 | ** | 0.0115 | * |
|  | Cultivar × Infestation | 1 | 1.8174 | 48.6000 | 0.0001 | *** | 0.0003 | *** |
|  | Cultivar × Sampling condition | 3 | 0.2760 | 7.3800 | 0.0007 | *** | 0.0020 | ** |
|  | Sampling condition × Infestation | 3 | 1.8084 | 48.3600 | 0.0001 | *** | 0.0003 | *** |
|  | Cultivar × Sampling condition × Infestation | 3 | 0.8231 | 22.0100 | 0.0001 | *** | 0.0003 | *** |
| Fumaric acid | Cultivar | 1 | 1892.0385 | 207.1600 | 0.0001 | *** | 0.0003 | *** |
|  | Infestation | 1 | 6.3075 | 0.6900 | 0.4121 | NS | 0.4838 | NS |
|  | Sampling condition | 3 | 1188.0556 | 130.0800 | 0.0001 | *** | 0.0003 | *** |
|  | Cultivar × Infestation | 1 | 12.6075 | 1.3800 | 0.2487 | NS | 0.3276 | NS |
|  | Cultivar × Sampling condition | 3 | 168.0321 | 18.4000 | 0.0001 | *** | 0.0003 | *** |
|  | Sampling condition × Infestation | 3 | 2097.7029 | 229.6800 | 0.0001 | *** | 0.0003 | *** |
|  | Cultivar × Sampling condition × Infestation | 3 | 85.5193 | 9.3600 | 0.0001 | *** | 0.0003 | *** |
| Galactonic acid | Cultivar | 1 | 8.1253 | 99.1400 | 0.0001 | *** | 0.0003 | *** |
|  | Infestation | 1 | 0.9355 | 11.4100 | 0.0020 | ** | 0.0054 | ** |
|  | Sampling condition | 3 | 0.1631 | 1.9900 | 0.1360 | NS | 0.2024 | NS |
|  | Cultivar × Infestation | 1 | 0.4960 | 6.0500 | 0.0197 | * | 0.0409 | * |
|  | Cultivar × Sampling condition | 3 | 0.1671 | 2.0400 | 0.1289 | NS | 0.1949 | NS |
|  | Sampling condition × Infestation | 3 | 0.4453 | 5.4300 | 0.0040 | ** | 0.0099 | ** |
|  | Cultivar × Sampling condition × Infestation | 3 | 0.0804 | 0.9800 | 0.4146 | NS | 0.4839 | NS |
| Gluconic acid 1,5-lactone | Cultivar | 1 | 5.1588 | 3.8000 | 0.0602 | NS | 0.1011 | NS |
|  | Infestation | 1 | 1.4323 | 1.0600 | 0.3121 | NS | 0.3894 | NS |
|  | Sampling condition | 3 | 4.3987 | 3.2400 | 0.0352 | * | 0.0662 | NS |
|  | Cultivar × Infestation | 1 | 40.4016 | 29.7900 | 0.0001 | *** | 0.0003 | *** |
|  | Cultivar × Sampling condition | 3 | 10.0252 | 7.3900 | 0.0007 | *** | 0.0020 | ** |
|  | Sampling condition × Infestation | 3 | 19.8283 | 14.6200 | 0.0001 | *** | 0.0003 | *** |
|  | Cultivar × Sampling condition × Infestation | 3 | 5.0133 | 3.7000 | 0.0220 | * | 0.0452 | * |
| Glycolic acid | Cultivar | 1 | 5.1169 | 0.9600 | 0.3357 | NS | 0.4133 | NS |
|  | Infestation | 1 | 3.7724 | 0.7000 | 0.4076 | NS | 0.4800 | NS |
|  | Sampling condition | 3 | 4.5127 | 0.8400 | 0.4807 | NS | 0.5388 | NS |
|  | Cultivar × Infestation | 1 | 5.8144 | 1.0900 | 0.3053 | NS | 0.3871 | NS |
|  | Cultivar × Sampling condition | 3 | 4.5010 | 0.8400 | 0.4818 | NS | 0.5388 | NS |
|  | Sampling condition × Infestation | 3 | 3.9372 | 0.7400 | 0.5387 | NS | 0.5972 | NS |
|  | Cultivar × Sampling condition × Infestation | 3 | 4.5333 | 0.8500 | 0.4787 | NS | 0.5385 | NS |
| Glyceric acid | Cultivar | 1 | 54.0601 | 2.9700 | 0.0943 | NS | 0.1498 | NS |
|  | Infestation | 1 | 297.7044 | 16.3700 | 0.0003 | *** | 0.0009 | *** |
|  | Sampling condition | 3 | 28.0798 | 1.5400 | 0.2221 | NS | 0.2998 | NS |
|  | Cultivar × Infestation | 1 | 192.0800 | 10.5600 | 0.0027 | ** | 0.0070 | ** |
|  | Cultivar × Sampling condition | 3 | 4.3896 | 0.2400 | 0.8668 | NS | 0.8981 | NS |
|  | Sampling condition × Infestation | 3 | 29.6356 | 1.6300 | 0.2019 | NS | 0.2765 | NS |
|  | Cultivar × Sampling condition × Infestation | 3 | 52.6039 | 2.8900 | 0.0505 | NS | 0.0880 | NS |
| Glyoxylic acid | Cultivar | 1 | 7.7683 | 65.1700 | 0.0001 | *** | 0.0003 | *** |
|  | Infestation | 1 | 24.5245 | 205.7400 | 0.0001 | *** | 0.0003 | *** |
|  | Sampling condition | 3 | 18.9239 | 158.7600 | 0.0001 | *** | 0.0003 | *** |
|  | Cultivar × Infestation | 1 | 1.1011 | 9.2400 | 0.0047 | ** | 0.0113 | * |
|  | Cultivar × Sampling condition | 3 | 3.0713 | 25.7700 | 0.0001 | *** | 0.0003 | *** |
|  | Sampling condition × Infestation | 3 | 6.1096 | 51.2500 | 0.0001 | *** | 0.0003 | *** |
|  | Cultivar × Sampling condition × Infestation | 3 | 2.9084 | 24.4000 | 0.0001 | *** | 0.0003 | *** |
| Lactic acid | Cultivar | 1 | 2.9164 | 0.7600 | 0.3909 | NS | 0.4632 | NS |
|  | Infestation | 1 | 1.4305 | 0.3700 | 0.5467 | NS | 0.6025 | NS |
|  | Sampling condition | 3 | 10.9175 | 2.8300 | 0.0543 | NS | 0.0933 | NS |
|  | Cultivar × Infestation | 1 | 6.2471 | 1.6200 | 0.2123 | NS | 0.2887 | NS |
|  | Cultivar × Sampling condition | 3 | 5.7974 | 1.5000 | 0.2327 | NS | 0.3119 | NS |
|  | Sampling condition × Infestation | 3 | 21.0567 | 5.4700 | 0.0039 | ** | 0.0098 | ** |
|  | Cultivar × Sampling condition × Infestation | 3 | 12.4451 | 3.2300 | 0.0357 | * | 0.0665 | NS |
| Lactic acid dimer | Cultivar | 1 | 6.6454 | 2.8900 | 0.0987 | NS | 0.1542 | NS |
|  | Infestation | 1 | 102.4336 | 44.5700 | 0.0001 | *** | 0.0003 | *** |
|  | Sampling condition | 3 | 165.1631 | 71.8700 | 0.0001 | *** | 0.0003 | *** |
|  | Cultivar × Infestation | 1 | 65.4734 | 28.4900 | 0.0001 | *** | 0.0003 | *** |
|  | Cultivar × Sampling condition | 3 | 10.7940 | 4.7000 | 0.0079 | ** | 0.0178 | * |
|  | Sampling condition × Infestation | 3 | 63.5798 | 27.6700 | 0.0001 | *** | 0.0003 | *** |
|  | Cultivar × Sampling condition × Infestation | 3 | 44.0866 | 19.1800 | 0.0001 | *** | 0.0003 | *** |
| Malic acid | Cultivar | 1 | 167.5895 | 5.0600 | 0.0315 | * | 0.0613 | NS |
|  | Infestation | 1 | 0.6651 | 0.0200 | 0.8882 | NS | 0.9123 | NS |
|  | Sampling condition | 3 | 608.8000 | 18.3800 | 0.0001 | *** | 0.0003 | *** |
|  | Cultivar × Infestation | 1 | 1.2578 | 0.0400 | 0.8467 | NS | 0.8841 | NS |
|  | Cultivar × Sampling condition | 3 | 78.4322 | 2.3700 | 0.0892 | NS | 0.1435 | NS |
|  | Sampling condition × Infestation | 3 | 236.8777 | 7.1500 | 0.0008 | *** | 0.0023 | ** |
|  | Cultivar × Sampling condition × Infestation | 3 | 94.9643 | 2.8700 | 0.0519 | NS | 0.0900 | NS |
| Malonic acid | Cultivar | 1 | 1482.3976 | 76.7800 | 0.0001 | *** | 0.0003 | *** |
|  | Infestation | 1 | 369.3555 | 19.1300 | 0.0001 | *** | 0.0003 | *** |
|  | Sampling condition | 3 | 363.9880 | 18.8500 | 0.0001 | *** | 0.0003 | *** |
|  | Cultivar × Infestation | 1 | 83.0710 | 4.3000 | 0.0464 | * | 0.0820 | NS |
|  | Cultivar × Sampling condition | 3 | 38.8694 | 2.0100 | 0.1325 | NS | 0.1986 | NS |
|  | Sampling condition × Infestation | 3 | 33.6435 | 1.7400 | 0.1787 | NS | 0.2520 | NS |
|  | Cultivar × Sampling condition × Infestation | 3 | 111.3850 | 5.7700 | 0.0030 | ** | 0.0077 | ** |
| Pyruvic acid | Cultivar | 1 | 10.2136 | 12.5500 | 0.0013 | ** | 0.0036 | ** |
|  | Infestation | 1 | 4.6215 | 5.6800 | 0.0235 | * | 0.0478 | * |
|  | Sampling condition | 3 | 5.1734 | 6.3600 | 0.0017 | ** | 0.0046 | ** |
|  | Cultivar × Infestation | 1 | 3.8668 | 4.7500 | 0.0370 | * | 0.0686 | NS |
|  | Cultivar × Sampling condition | 3 | 4.4701 | 5.4900 | 0.0038 | ** | 0.0096 | ** |
|  | Sampling condition × Infestation | 3 | 0.9487 | 1.1700 | 0.3386 | NS | 0.4156 | NS |
|  | Cultivar × Sampling condition × Infestation | 3 | 0.0754 | 0.0900 | 0.9635 | NS | 0.9665 | NS |
| Succinic acid | Cultivar | 1 | 2.3725 | 2.0600 | 0.1614 | NS | 0.2329 | NS |
|  | Infestation | 1 | 63.1483 | 54.8800 | 0.0001 | *** | 0.0003 | *** |
|  | Sampling condition | 3 | 45.4371 | 39.4900 | 0.0001 | *** | 0.0003 | *** |
|  | Cultivar × Infestation | 1 | 1.2393 | 1.0800 | 0.3077 | NS | 0.3871 | NS |
|  | Cultivar × Sampling condition | 3 | 11.2445 | 9.7700 | 0.0001 | *** | 0.0003 | *** |
|  | Sampling condition × Infestation | 3 | 8.1846 | 7.1100 | 0.0010 | ** | 0.0028 | ** |
|  | Cultivar × Sampling condition × Infestation | 3 | 1.6917 | 1.4700 | 0.2425 | NS | 0.3228 | NS |
| Tartaric acid | Cultivar | 1 | 28.4130 | 27.3400 | 0.0001 | *** | 0.0003 | *** |
|  | Infestation | 1 | 0.5786 | 0.5600 | 0.4610 | NS | 0.5221 | NS |
|  | Sampling condition | 3 | 7.2945 | 7.0200 | 0.0009 | *** | 0.0026 | ** |
|  | Cultivar × Infestation | 1 | 0.0023 | 0.0000 | 0.9630 | NS | 0.9665 | NS |
|  | Cultivar × Sampling condition | 3 | 1.4141 | 1.3600 | 0.2724 | NS | 0.3533 | NS |
|  | Sampling condition × Infestation | 3 | 2.0852 | 2.0100 | 0.1329 | NS | 0.1986 | NS |
|  | Cultivar × Sampling condition × Infestation | 3 | 2.0157 | 1.9400 | 0.1431 | NS | 0.2097 | NS |
| Threonic acid | Cultivar | 1 | 28.4438 | 26.3400 | 0.0001 | *** | 0.0003 | *** |
|  | Infestation | 1 | 23.5620 | 21.8200 | 0.0001 | *** | 0.0003 | *** |
|  | Sampling condition | 3 | 9.3052 | 8.6200 | 0.0002 | *** | 0.0007 | *** |
|  | Cultivar × Infestation | 1 | 4.0892 | 3.7900 | 0.0605 | NS | 0.1012 | NS |
|  | Cultivar × Sampling condition | 3 | 2.0447 | 1.8900 | 0.1505 | NS | 0.2188 | NS |
|  | Sampling condition × Infestation | 3 | 10.7687 | 9.9700 | 0.0001 | *** | 0.0003 | *** |
|  | Cultivar × Sampling condition × Infestation | 3 | 10.0845 | 9.3400 | 0.0001 | *** | 0.0003 | *** |
| **Phenolics** | | | | | | | | |
| Salicylic acid | Cultivar | 1 | 1.8058 | 83.6100 | 0.0001 | *** | 0.0003 | *** |
|  | Infestation | 1 | 3.0351 | 140.5300 | 0.0001 | *** | 0.0003 | *** |
|  | Sampling condition | 3 | 10.4814 | 485.2900 | 0.0001 | *** | 0.0003 | *** |
|  | Cultivar × Infestation | 1 | 5.1549 | 238.6700 | 0.0001 | *** | 0.0003 | *** |
|  | Cultivar × Sampling condition | 3 | 1.2477 | 57.7700 | 0.0001 | *** | 0.0003 | *** |
|  | Sampling condition × Infestation | 3 | 9.0059 | 416.9800 | 0.0001 | *** | 0.0003 | *** |
|  | Cultivar × Sampling condition × Infestation | 3 | 3.7900 | 175.4800 | 0.0001 | *** | 0.0003 | *** |
| 2-Hydroxycinnamic acid | Cultivar | 1 | 5.9855 | 15.8600 | 0.0004 | *** | 0.0012 | ** |
|  | Infestation | 1 | 6.7275 | 17.8300 | 0.0002 | *** | 0.0007 | *** |
|  | Sampling condition | 3 | 10.8596 | 28.7800 | 0.0001 | *** | 0.0003 | *** |
|  | Cultivar × Infestation | 1 | 0.8614 | 2.2800 | 0.1406 | NS | 0.2080 | NS |
|  | Cultivar × Sampling condition | 3 | 1.1955 | 3.1700 | 0.0376 | * | 0.0693 | NS |
|  | Sampling condition × Infestation | 3 | 4.7934 | 12.7000 | 0.0001 | *** | 0.0003 | *** |
|  | Cultivar × Sampling condition × Infestation | 3 | 1.3591 | 3.6000 | 0.0239 | * | 0.0483 | * |
| 4-Hydroxycinnamic acid | Cultivar | 1 | 0.1009 | 1.8300 | 0.1855 | NS | 0.2597 | NS |
|  | Infestation | 1 | 0.2833 | 5.1500 | 0.0304 | * | 0.0598 | NS |
|  | Sampling condition | 3 | 1.2011 | 21.8300 | 0.0001 | *** | 0.0003 | *** |
|  | Cultivar × Infestation | 1 | 0.1403 | 2.5500 | 0.1205 | NS | 0.1855 | NS |
|  | Cultivar × Sampling condition | 3 | 0.1690 | 3.0700 | 0.0422 | * | 0.0767 | NS |
|  | Sampling condition × Infestation | 3 | 0.2190 | 3.9800 | 0.0165 | * | 0.0350 | * |
|  | Cultivar × Sampling condition × Infestation | 3 | 0.0687 | 1.2500 | 0.3093 | NS | 0.3871 | NS |
| Quercetin | Cultivar | 1 | 21.4501 | 0.0800 | 0.7841 | NS | 0.8279 | NS |
|  | Infestation | 1 | 151.1740 | 0.5400 | 0.4688 | NS | 0.5290 | NS |
|  | Sampling condition | 3 | 355.5340 | 1.2700 | 0.3033 | NS | 0.3860 | NS |
|  | Cultivar × Infestation | 1 | 2866.9534 | 10.2000 | 0.0032 | ** | 0.0081 | ** |
|  | Cultivar × Sampling condition | 3 | 273.9151 | 0.9700 | 0.4171 | NS | 0.4851 | NS |
|  | Sampling condition × Infestation | 3 | 939.8955 | 3.3500 | 0.0316 | * | 0.0613 | NS |
|  | Cultivar × Sampling condition × Infestation | 3 | 180.1314 | 0.6400 | 0.5944 | NS | 0.6494 | NS |
| **Inorganic acid** |  |  |  |  |  |  |  |  |
| Phosphoric acid | Cultivar | 1 | 151.1915 | 0.0600 | 0.8108 | NS | 0.8490 | NS |
|  | Infestation | 1 | 22660.8887 | 8.7400 | 0.0059 | ** | 0.0139 | * |
|  | Sampling condition | 3 | 44555.5506 | 17.1800 | 0.0001 | *** | 0.0003 | *** |
|  | Cultivar × Infestation | 1 | 3414.3724 | 1.3200 | 0.2601 | NS | 0.3390 | NS |
|  | Cultivar × Sampling condition | 3 | 5259.1212 | 2.0300 | 0.1305 | NS | 0.1965 | NS |
|  | Sampling condition × Infestation | 3 | 21657.0756 | 8.3500 | 0.0003 | *** | 0.0009 | *** |
|  | Cultivar × Sampling condition × Infestation | 3 | 7719.0584 | 2.9800 | 0.0467 | * | 0.0821 | NS |
| **Amino acids** | | | | | | | | |
| β-Alanine | Cultivar | 1 | 2.3585 | 32.9200 | 0.0001 | *** | 0.0003 | *** |
|  | Infestation | 1 | 2.4031 | 33.5500 | 0.0001 | *** | 0.0003 | *** |
|  | Sampling condition | 3 | 0.3757 | 5.2500 | 0.0047 | ** | 0.0113 | * |
|  | Cultivar × Infestation | 1 | 1.5480 | 21.6100 | 0.0001 | *** | 0.0003 | *** |
|  | Cultivar × Sampling condition | 3 | 0.0476 | 0.6600 | 0.5803 | NS | 0.6358 | NS |
|  | Sampling condition × Infestation | 3 | 2.1709 | 30.3000 | 0.0001 | *** | 0.0003 | *** |
|  | Cultivar × Sampling condition × Infestation | 3 | 0.1720 | 2.4000 | 0.0859 | NS | 0.1388 | NS |
| Glycine | Cultivar | 1 | 2080.6638 | 1.3400 | 0.2557 | NS | 0.3356 | NS |
|  | Infestation | 1 | 1842.4440 | 1.1900 | 0.2843 | NS | 0.3668 | NS |
|  | Sampling condition | 3 | 1368.0448 | 0.8800 | 0.4613 | NS | 0.5221 | NS |
|  | Cultivar × Infestation | 1 | 1489.0815 | 0.9600 | 0.3349 | NS | 0.4133 | NS |
|  | Cultivar × Sampling condition | 3 | 1458.8777 | 0.9400 | 0.4331 | NS | 0.4961 | NS |
|  | Sampling condition × Infestation | 3 | 1725.1093 | 1.1100 | 0.3593 | NS | 0.4367 | NS |
|  | Cultivar × Sampling condition × Infestation | 3 | 1520.8942 | 0.9800 | 0.4148 | NS | 0.4839 | NS |
| Glycine (3TMS derivative) | Cultivar | 1 | 10.7686 | 17.2300 | 0.0003 | *** | 0.0009 | *** |
|  | Infestation | 1 | 22.1826 | 35.4900 | 0.0001 | *** | 0.0003 | *** |
|  | Sampling condition | 3 | 22.6523 | 36.2400 | 0.0001 | *** | 0.0003 | *** |
|  | Cultivar × Infestation | 1 | 17.8299 | 28.5200 | 0.0001 | *** | 0.0003 | *** |
|  | Cultivar × Sampling condition | 3 | 4.5766 | 7.3200 | 0.0008 | *** | 0.0023 | ** |
|  | Sampling condition × Infestation | 3 | 16.4442 | 26.3100 | 0.0001 | *** | 0.0003 | *** |
|  | Cultivar × Sampling condition × Infestation | 3 | 11.6481 | 18.6300 | 0.0001 | *** | 0.0003 | *** |
| Phenylalanine | Cultivar | 1 | 0.1292 | 0.0300 | 0.8732 | NS | 0.9018 | NS |
|  | Infestation | 1 | 46.5511 | 9.3300 | 0.0045 | ** | 0.0110 | * |
|  | Sampling condition | 3 | 74.1717 | 14.8700 | 0.0001 | *** | 0.0003 | *** |
|  | Cultivar × Infestation | 1 | 0.9776 | 0.2000 | 0.6610 | NS | 0.7139 | NS |
|  | Cultivar × Sampling condition | 3 | 8.2119 | 1.6500 | 0.1983 | NS | 0.2726 | NS |
|  | Sampling condition × Infestation | 3 | 66.6048 | 13.3500 | 0.0001 | *** | 0.0003 | *** |
|  | Cultivar × Sampling condition × Infestation | 3 | 2.2097 | 0.4400 | 0.7239 | NS | 0.7686 | NS |
| Serine (2TMS derivative) | Cultivar | 1 | 212.1233 | 8.6300 | 0.0062 | ** | 0.0145 |  |
|  | Infestation | 1 | 86.8041 | 3.5300 | 0.0696 | NS | 0.1149 |  |
|  | Sampling condition | 3 | 341.8571 | 13.9100 | 0.0001 | *** | 0.0003 |  |
|  | Cultivar × Infestation | 1 | 356.5479 | 14.5100 | 0.0006 | *** | 0.0018 |  |
|  | Cultivar × Sampling condition | 3 | 430.0279 | 17.5000 | 0.0001 | *** | 0.0003 |  |
|  | Sampling condition × Infestation | 3 | 554.0527 | 22.5400 | 0.0001 | *** | 0.0003 |  |
|  | Cultivar × Sampling condition × Infestation | 3 | 162.9192 | 6.6300 | 0.0014 | ** | 0.0038 |  |
| Serine (3TMS derivative) | Cultivar | 1 | 6.9998 | 0.1900 | 0.6685 | NS | 0.7199 |  |
|  | Infestation | 1 | 30.3849 | 0.8100 | 0.3746 | NS | 0.4524 |  |
|  | Sampling condition | 3 | 100.7929 | 2.6900 | 0.0628 | NS | 0.1046 |  |
|  | Cultivar × Infestation | 1 | 291.4124 | 7.7800 | 0.0088 | ** | 0.0195 |  |
|  | Cultivar × Sampling condition | 3 | 228.9839 | 6.1100 | 0.0021 | ** | 0.0056 |  |
|  | Sampling condition × Infestation | 3 | 333.4971 | 8.9000 | 0.0002 | *** | 0.0007 |  |
|  | Cultivar × Sampling condition × Infestation | 3 | 58.4051 | 1.5600 | 0.2186 | NS | 0.2962 |  |
| Threonine | Cultivar | 1 | 2.4390 | 4.2700 | 0.0470 | * | 0.0823 | NS |
|  | Infestation | 1 | 1.4077 | 2.4600 | 0.1263 | NS | 0.1923 | NS |
|  | Sampling condition | 3 | 7.7040 | 13.4900 | 0.0001 | *** | 0.0003 | *** |
|  | Cultivar × Infestation | 1 | 10.7541 | 18.8300 | 0.0001 | *** | 0.0003 | *** |
|  | Cultivar × Sampling condition | 3 | 3.8213 | 6.6900 | 0.0012 | ** | 0.0033 | ** |
|  | Sampling condition × Infestation | 3 | 2.3328 | 4.0800 | 0.0146 | * | 0.0317 | * |
|  | Cultivar × Sampling condition × Infestation | 3 | 7.1643 | 12.5400 | 0.0001 | *** | 0.0003 | *** |
| Valine (2TMS derivative) | Cultivar | 1 | 63.5913 | 2.9700 | 0.0947 | NS | 0.1498 | NS |
|  | Infestation | 1 | 4.1978 | 0.2000 | 0.6610 | NS | 0.7139 | NS |
|  | Sampling condition | 3 | 114.1314 | 5.3300 | 0.0044 | ** | 0.0108 | * |
|  | Cultivar × Infestation | 1 | 46.3227 | 2.1600 | 0.1514 | NS | 0.2193 | NS |
|  | Cultivar × Sampling condition | 3 | 59.9729 | 2.8000 | 0.0562 | NS | 0.0957 | NS |
|  | Sampling condition × Infestation | 3 | 205.8347 | 9.6200 | 0.0001 | *** | 0.0003 | *** |
|  | Cultivar × Sampling condition × Infestation | 3 | 65.9632 | 3.0800 | 0.0417 | * | 0.0761 | NS |
| **Sugars** | | | | | | | | |
| Arabinose | Cultivar | 1 | 0.1633 | 1.3800 | 0.2480 | NS | 0.3276 | NS |
|  | Infestation | 1 | 0.4641 | 3.9400 | 0.0559 | NS | 0.0956 | NS |
|  | Sampling condition | 3 | 1.7869 | 15.1500 | 0.0001 | *** | 0.0003 | *** |
|  | Cultivar × Infestation | 1 | 0.0280 | 0.2400 | 0.6292 | NS | 0.6854 | NS |
|  | Cultivar × Sampling condition | 3 | 0.2072 | 1.7600 | 0.1753 | NS | 0.2501 | NS |
|  | Sampling condition × Infestation | 3 | 2.5820 | 21.8900 | 0.0001 | *** | 0.0003 | *** |
|  | Cultivar × Sampling condition × Infestation | 3 | 0.1246 | 1.0600 | 0.3811 | NS | 0.4559 | NS |
| Fructose | Cultivar | 1 | 522.5880 | 1.9400 | 0.1730 | NS | 0.2477 | NS |
|  | Infestation | 1 | 2219.2480 | 8.2500 | 0.0072 | ** | 0.0164 | * |
|  | Sampling condition | 3 | 667.5564 | 2.4800 | 0.0788 | NS | 0.1284 | NS |
|  | Cultivar × Infestation | 1 | 1694.3257 | 6.3000 | 0.0174 | * | 0.0367 | * |
|  | Cultivar × Sampling condition | 3 | 107.1813 | 0.4000 | 0.7551 | NS | 0.7995 | NS |
|  | Sampling condition × Infestation | 3 | 5569.9709 | 20.7000 | 0.0001 | *** | 0.0003 | *** |
|  | Cultivar × Sampling condition × Infestation | 3 | 769.2142 | 2.8600 | 0.0523 | NS | 0.0903 | NS |
| Galactose | Cultivar | 1 | 0.0165 | 0.0200 | 0.9023 | NS | 0.9243 | NS |
|  | Infestation | 1 | 4.6563 | 4.3200 | 0.0458 | * | 0.0817 | NS |
|  | Sampling condition | 3 | 1.3457 | 1.2500 | 0.3085 | NS | 0.3871 | NS |
|  | Cultivar × Infestation | 1 | 5.8172 | 5.4000 | 0.0267 | * | 0.0537 | NS |
|  | Cultivar × Sampling condition | 3 | 3.2053 | 2.9700 | 0.0463 | * | 0.0820 | NS |
|  | Sampling condition × Infestation | 3 | 0.2865 | 0.2700 | 0.8496 | NS | 0.8847 | NS |
|  | Cultivar × Sampling condition × Infestation | 3 | 3.7228 | 3.4500 | 0.0278 | * | 0.0553 | NS |
| Glucoheptose | Cultivar | 1 | 0.0029 | 0.4300 | 0.5168 | NS | 0.5746 | NS |
|  | Infestation | 1 | 0.0001 | 0.0100 | 0.9182 | NS | 0.9330 | NS |
|  | Sampling condition | 3 | 0.0308 | 4.5400 | 0.0095 | ** | 0.0209 | * |
|  | Cultivar × Infestation | 1 | 0.0063 | 0.9300 | 0.3413 | NS | 0.4175 | NS |
|  | Cultivar × Sampling condition | 3 | 0.0158 | 2.3400 | 0.0930 | NS | 0.1483 | NS |
|  | Sampling condition × Infestation | 3 | 0.0226 | 3.3300 | 0.0322 | * | 0.0620 | NS |
|  | Cultivar × Sampling condition × Infestation | 3 | 0.0212 | 3.1300 | 0.0396 | * | 0.0727 | NS |
| Glucose | Cultivar | 1 | 237.1408 | 5.2600 | 0.0286 | * | 0.0566 | NS |
|  | Infestation | 1 | 100.0808 | 2.2200 | 0.1461 | NS | 0.2132 | NS |
|  | Sampling condition | 3 | 51.7482 | 1.1500 | 0.3449 | NS | 0.4206 | NS |
|  | Cultivar × Infestation | 1 | 217.6434 | 4.8300 | 0.0354 | * | 0.0662 | NS |
|  | Cultivar × Sampling condition | 3 | 21.6660 | 0.4800 | 0.6982 | NS | 0.7476 | NS |
|  | Sampling condition × Infestation | 3 | 903.9086 | 20.0400 | 0.0001 | *** | 0.0003 | *** |
|  | Cultivar × Sampling condition × Infestation | 3 | 68.2402 | 1.5100 | 0.2299 | NS | 0.3093 | NS |
| Isomaltose | Cultivar | 1 | 7.8880 | 4.4700 | 0.0425 | * | 0.0769 | NS |
|  | Infestation | 1 | 1.3650 | 0.7700 | 0.3857 | NS | 0.4599 | NS |
|  | Sampling condition | 3 | 83.7423 | 47.5000 | 0.0001 | *** | 0.0003 | *** |
|  | Cultivar × Infestation | 1 | 2.0364 | 1.1600 | 0.2908 | NS | 0.3739 | NS |
|  | Cultivar × Sampling condition | 3 | 4.1723 | 2.3700 | 0.0899 | NS | 0.1440 | NS |
|  | Sampling condition × Infestation | 3 | 73.7220 | 41.8200 | 0.0001 | *** | 0.0003 | *** |
|  | Cultivar × Sampling condition × Infestation | 3 | 0.8252 | 0.4700 | 0.7066 | NS | 0.7545 | NS |
| Lyxose | Cultivar | 1 | 1.9521 | 8.1900 | 0.0074 | ** | 0.0167 | * |
|  | Infestation | 1 | 2.6414 | 11.0800 | 0.0022 | ** | 0.0058 | ** |
|  | Sampling condition | 3 | 1.4761 | 6.1900 | 0.0019 | ** | 0.0051 | ** |
|  | Cultivar × Infestation | 1 | 1.4214 | 5.9600 | 0.0203 | * | 0.0419 | * |
|  | Cultivar × Sampling condition | 3 | 0.0223 | 0.0900 | 0.9630 | NS | 0.9665 | NS |
|  | Sampling condition × Infestation | 3 | 1.3581 | 5.7000 | 0.0030 | ** | 0.0077 | ** |
|  | Cultivar × Sampling condition × Infestation | 3 | 0.7260 | 3.0500 | 0.0428 | * | 0.0770 | NS |
| Maltose | Cultivar | 1 | 4.4221 | 0.3500 | 0.5568 | NS | 0.6118 | NS |
|  | Infestation | 1 | 48.5942 | 3.8800 | 0.0579 | NS | 0.0979 | NS |
|  | Sampling condition | 3 | 119.7803 | 9.5600 | 0.0001 | *** | 0.0003 | *** |
|  | Cultivar × Infestation | 1 | 17.7062 | 1.4100 | 0.2436 | NS | 0.3231 | NS |
|  | Cultivar × Sampling condition | 3 | 4.3082 | 0.3400 | 0.7938 | NS | 0.8358 | NS |
|  | Sampling condition × Infestation | 3 | 238.2801 | 19.0200 | 0.0001 | *** | 0.0003 | *** |
|  | Cultivar × Sampling condition × Infestation | 3 | 13.5570 | 1.0800 | 0.3712 | NS | 0.4497 | NS |
| Maltotriose | Cultivar | 1 | 2534.0685 | 5.1000 | 0.0312 | * | 0.0611 | NS |
|  | Infestation | 1 | 8101.6342 | 16.2900 | 0.0003 | *** | 0.0009 | *** |
|  | Sampling condition | 3 | 737.7236 | 1.4800 | 0.2383 | NS | 0.3183 | NS |
|  | Cultivar × Infestation | 1 | 8733.7252 | 17.5600 | 0.0002 | *** | 0.0007 | *** |
|  | Cultivar × Sampling condition | 3 | 2469.3694 | 4.9700 | 0.0063 | ** | 0.0146 | * |
|  | Sampling condition × Infestation | 3 | 7901.1392 | 15.8900 | 0.0001 | *** | 0.0003 | *** |
|  | Cultivar × Sampling condition × Infestation | 3 | 2299.7162 | 4.6200 | 0.0087 | ** | 0.0193 | * |
| Mannose | Cultivar | 1 | 0.1160 | 5.7200 | 0.0228 | * | 0.0466 | * |
|  | Infestation | 1 | 0.0675 | 3.3300 | 0.0774 | NS | 0.1267 | NS |
|  | Sampling condition | 3 | 0.0466 | 2.3000 | 0.0962 | NS | 0.1509 | NS |
|  | Cultivar × Infestation | 1 | 0.1728 | 8.5200 | 0.0064 | ** | 0.0148 | * |
|  | Cultivar × Sampling condition | 3 | 0.0147 | 0.7200 | 0.5451 | NS | 0.6025 | NS |
|  | Sampling condition × Infestation | 3 | 0.4859 | 23.9700 | 0.0001 | *** | 0.0003 | *** |
|  | Cultivar × Sampling condition × Infestation | 3 | 0.0395 | 1.9500 | 0.1417 | NS | 0.2084 | NS |
| Ribose | Cultivar | 1 | 4.5880 | 3.5500 | 0.0687 | NS | 0.1139 | NS |
|  | Infestation | 1 | 12.1203 | 9.3700 | 0.0044 | ** | 0.0108 | * |
|  | Sampling condition | 3 | 15.1409 | 11.7100 | 0.0001 | *** | 0.0003 | *** |
|  | Cultivar × Infestation | 1 | 2.5947 | 2.0100 | 0.1663 | NS | 0.2390 | NS |
|  | Cultivar × Sampling condition | 3 | 4.2513 | 3.2900 | 0.0331 | * | 0.0632 | NS |
|  | Sampling condition × Infestation | 3 | 23.6441 | 18.2900 | 0.0001 | *** | 0.0003 | *** |
|  | Cultivar × Sampling condition × Infestation | 3 | 9.9425 | 7.6900 | 0.0005 | *** | 0.0015 | ** |
| Rhamnose | Cultivar | 1 | 0.1938 | 8.8000 | 0.0057 | ** | 0.0136 | * |
|  | Infestation | 1 | 0.0029 | 0.1300 | 0.7213 | NS | 0.7680 | NS |
|  | Sampling condition | 3 | 0.3418 | 15.5300 | 0.0001 | *** | 0.0003 | *** |
|  | Cultivar × Infestation | 1 | 0.0144 | 0.6500 | 0.4254 | NS | 0.4902 | NS |
|  | Cultivar × Sampling condition | 3 | 0.0430 | 1.9500 | 0.1409 | NS | 0.2080 | NS |
|  | Sampling condition × Infestation | 3 | 0.2576 | 11.7000 | 0.0001 | *** | 0.0003 | *** |
|  | Cultivar × Sampling condition × Infestation | 3 | 0.0466 | 2.1200 | 0.1176 | NS | 0.1822 | NS |
| 4-Aminobutyric acid (GABA) | Cultivar | 1 | 53786.2910 | 135.4600 | 0.0001 | *** | 0.0003 | *** |
|  | Infestation | 1 | 9850.4430 | 24.8100 | 0.0001 | *** | 0.0003 | *** |
|  | Sampling condition | 3 | 6664.8106 | 16.7900 | 0.0001 | *** | 0.0003 | *** |
|  | Cultivar × Infestation | 1 | 5785.5817 | 14.5700 | 0.0006 | *** | 0.0018 | ** |
|  | Cultivar × Sampling condition | 3 | 831.0507 | 2.0900 | 0.1207 | NS | 0.1855 | NS |
|  | Sampling condition × Infestation | 3 | 16534.5768 | 41.6400 | 0.0001 | *** | 0.0003 | *** |
|  | Cultivar × Sampling condition × Infestation | 3 | 1978.7886 | 4.9800 | 0.0060 | ** | 0.0141 | * |
| Aspartic acid | Cultivar | 1 | 333.0640 | 108.8000 | 0.0001 | *** | 0.0003 | *** |
|  | Infestation | 1 | 56.2034 | 18.3600 | 0.0002 | *** | 0.0007 | *** |
|  | Sampling condition | 3 | 163.0524 | 53.2600 | 0.0001 | *** | 0.0003 | *** |
|  | Cultivar × Infestation | 1 | 20.9616 | 6.8500 | 0.0134 | * | 0.0293 | * |
|  | Cultivar × Sampling condition | 3 | 11.5934 | 3.7900 | 0.0197 | * | 0.0409 | * |
|  | Sampling condition × Infestation | 3 | 91.4817 | 29.8800 | 0.0001 | *** | 0.0003 | *** |
|  | Cultivar × Sampling condition × Infestation | 3 | 86.0339 | 28.1000 | 0.0001 | *** | 0.0003 | *** |
| **Sugar alcohols (Polyols)** | | | | | | | | |
| Allo-inositol | Cultivar | 1 | 5.5803 | 0.1800 | 0.6776 | NS | 0.7276 | NS |
|  | Infestation | 1 | 20.0455 | 0.6300 | 0.4324 | NS | 0.4961 | NS |
|  | Sampling condition | 3 | 398.6451 | 12.5800 | 0.0001 | *** | 0.0003 | *** |
|  | Cultivar × Infestation | 1 | 2.1349 | 0.0700 | 0.7969 | NS | 0.8367 | NS |
|  | Cultivar × Sampling condition | 3 | 55.5089 | 1.7500 | 0.1769 | NS | 0.2514 | NS |
|  | Sampling condition × Infestation | 3 | 401.1522 | 12.6600 | 0.0001 | *** | 0.0003 | *** |
|  | Cultivar × Sampling condition × Infestation | 3 | 153.8503 | 4.8600 | 0.0070 | ** | 0.0160 | * |
| Mannitol | Cultivar | 1 | 349.2321 | 282.2100 | 0.0001 | *** | 0.0003 | *** |
|  | Infestation | 1 | 3.4775 | 2.8100 | 0.1037 | NS | 0.1613 | NS |
|  | Sampling condition | 3 | 47.0481 | 38.0200 | 0.0001 | *** | 0.0003 | *** |
|  | Cultivar × Infestation | 1 | 0.0352 | 0.0300 | 0.8672 | NS | 0.8981 | NS |
|  | Cultivar × Sampling condition | 3 | 27.3984 | 22.1400 | 0.0001 | *** | 0.0003 | *** |
|  | Sampling condition × Infestation | 3 | 45.2306 | 36.5500 | 0.0001 | *** | 0.0003 | *** |
|  | Cultivar × Sampling condition × Infestation | 3 | 29.4028 | 23.7600 | 0.0001 | *** | 0.0003 | *** |
| Xylitol | Cultivar | 1 | 14.0076 | 16.2300 | 0.0003 | *** | 0.0009 | *** |
|  | Infestation | 1 | 12.4339 | 14.4100 | 0.0006 | *** | 0.0018 | ** |
|  | Sampling condition | 3 | 5.7678 | 6.6800 | 0.0012 | ** | 0.0033 | ** |
|  | Cultivar × Infestation | 1 | 1.1501 | 1.3300 | 0.2569 | NS | 0.3360 | NS |
|  | Cultivar × Sampling condition | 3 | 2.7895 | 3.2300 | 0.0351 | * | 0.0662 | NS |
|  | Sampling condition × Infestation | 3 | 7.0594 | 8.1800 | 0.0003 | *** | 0.0009 | *** |
|  | Cultivar × Sampling condition × Infestation | 3 | 1.1728 | 1.3600 | 0.2729 | NS | 0.3533 | NS |
| **Polyamines** | | | | | | | | |
| Spermidine | Cultivar | 1 | 0.1111 | 1.0000 | 0.3243 | NS | 0.4032 | NS |
|  | Infestation | 1 | 1.1561 | 10.4400 | 0.0029 | ** | 0.0075 | ** |
|  | Sampling condition | 3 | 4.9168 | 44.4000 | 0.0001 | *** | 0.0003 | *** |
|  | Cultivar × Infestation | 1 | 0.3504 | 3.1600 | 0.0851 | NS | 0.1381 | NS |
|  | Cultivar × Sampling condition | 3 | 0.0253 | 0.2300 | 0.8762 | NS | 0.9025 | NS |
|  | Sampling condition × Infestation | 3 | 2.3862 | 21.5500 | 0.0001 | *** | 0.0003 | *** |
|  | Cultivar × Sampling condition × Infestation | 3 | 0.1178 | 1.0600 | 0.3788 | NS | 0.4546 | NS |
| **Sugar phosphates** | | | | | | | | |
| Glycerol-3-phosphate | Cultivar | 1 | 4.7565 | 1.8200 | 0.1871 | NS | 0.2610 | NS |
|  | Infestation | 1 | 2.0959 | 0.8000 | 0.3776 | NS | 0.4546 | NS |
|  | Sampling condition | 3 | 19.2985 | 7.3700 | 0.0007 | *** | 0.0020 | ** |
|  | Cultivar × Infestation | 1 | 1.5660 | 0.6000 | 0.4449 | NS | 0.5081 | NS |
|  | Cultivar × Sampling condition | 3 | 2.3511 | 0.9000 | 0.4528 | NS | 0.5155 | NS |
|  | Sampling condition × Infestation | 3 | 4.1583 | 1.5900 | 0.2114 | NS | 0.2885 | NS |
|  | Cultivar × Sampling condition × Infestation | 3 | 4.4159 | 1.6900 | 0.1894 | NS | 0.2632 | NS |
| Ribose-5-phosphate | Cultivar | 1 | 2.8825 | 6.2200 | 0.0182 | * | 0.0382 | * |
|  | Infestation | 1 | 1.3700 | 2.9500 | 0.0956 | NS | 0.1506 | NS |
|  | Sampling condition | 3 | 1.1944 | 2.5800 | 0.0717 | NS | 0.1178 | NS |
|  | Cultivar × Infestation | 1 | 0.0062 | 0.0100 | 0.9085 | NS | 0.9281 | NS |
|  | Cultivar × Sampling condition | 3 | 0.4455 | 0.9600 | 0.4236 | NS | 0.4897 | NS |
|  | Sampling condition × Infestation | 3 | 1.8912 | 4.0800 | 0.0150 | * | 0.0324 | * |
|  | Cultivar × Sampling condition × Infestation | 3 | 0.9525 | 2.0500 | 0.1267 | NS | 0.1923 | NS |
| **Nitrogenous bases** |  |  |  |  |  |  |  |  |
| Adenine | Cultivar | 1 | 0.0027 | 0.0100 | 0.9407 | NS | 0.9533 | NS |
|  | Infestation | 1 | 0.4681 | 0.9700 | 0.3311 | NS | 0.4103 | NS |
|  | Sampling condition | 3 | 4.7084 | 9.8000 | 0.0001 | *** | 0.0003 | *** |
|  | Cultivar × Infestation | 1 | 0.3571 | 0.7400 | 0.3951 | NS | 0.4667 | NS |
|  | Cultivar × Sampling condition | 3 | 0.4995 | 1.0400 | 0.3883 | NS | 0.4616 | NS |
|  | Sampling condition × Infestation | 3 | 8.7189 | 18.1500 | 0.0001 | *** | 0.0003 | *** |
|  | Cultivar × Sampling condition × Infestation | 3 | 1.4515 | 3.0200 | 0.0440 | * | 0.0788 | NS |
| **Amides** | | | | | | | | |
| Urea | Cultivar | 1 | 14.8092 | 1.1100 | 0.2999 | NS | 0.3830 | NS |
|  | Infestation | 1 | 65.7400 | 4.9300 | 0.0338 | * | 0.0642 | NS |
|  | Sampling condition | 3 | 23.0881 | 1.7300 | 0.1807 | NS | 0.2539 | NS |
|  | Cultivar × Infestation | 1 | 51.6121 | 3.8700 | 0.0580 | NS | 0.0979 | NS |
|  | Cultivar × Sampling condition | 3 | 22.3513 | 1.6800 | 0.1921 | NS | 0.2658 | NS |
|  | Sampling condition × Infestation | 3 | 16.9970 | 1.2800 | 0.2999 | NS | 0.3830 | NS |
|  | Cultivar × Sampling condition × Infestation | 3 | 1.2246 | 0.0900 | 0.9639 | NS | 0.9665 | NS |

Note: The table contains 54 metabolites and 378 ANOVA effect rows.
